# Challenges in Using the Official Italian Method to Detect Bovine Whey Proteins in Protected Designation of Origin Buffalo Mozzarella: A Proteomic Approach to Face Observed Limits

**DOI:** 10.3390/foods14050822

**Published:** 2025-02-27

**Authors:** Federica Della Cerra, Mariapia Esposito, Simonetta Caira, Andrea Scaloni, Francesco Addeo

**Affiliations:** 1 Proteomics, Metabolomics & Mass Spectrometry Laboratory, Institute for the Animal Production System in the Mediterranean Environment, National Research Council, 80055 Portici, Italy; federicadellacerra@cnr.it (F.D.C.); mariaesposito@cnr.it (M.E.); andrea.scaloni@cnr.it (A.S.); 2 Department of Agriculture, University of Naples “Federico II”, 80055 Portici, Italy; doglie42@gmail.com

**Keywords:** buffalo milk adulteration, buffalo mozzarella (PDO), MALDI-TOF-MS, nano-HPLC-ESI-MS/MS, proteomics, β-lactoglobulin, food authenticity

## Abstract

This study critically examines the limitations of the official Italian methodology used for detecting bovine adulteration milk in Protected Designation of Origin (PDO) Mozzarella di Bufala Campana (MdBC). This method focuses on the whey fraction of cheese samples, which comprises about 1% of total MdBC proteins, and is based on a high-performance liquid chromatography (HPLC) quantification of the bovine β-lactoglobulin A (β-Lg A) as a marker. Here, we have demonstrated that this official methodology suffers from measurement inconsistencies due to its reliance on raw bovine whey standards, which fail to account for β-Lg genetic polymorphisms in real MdBC samples and protein thermal modifications during cheesemaking. To overcome these limitations, we propose a dual proteomics-based approach using matrix-assisted laser desorption ionization (MALDI-TOF) mass spectrometry (MS) and nano-HPLC-electrospray (ESI)−tandem mass spectrometry (MS/MS) analysis of MdBC extracted whey. MALDI-TOF-MS focused on identifying proteotypic peptides specific to bovine and buffalo β-Lg and α-lactalbumin (α-La), enabling high specificity for distinguishing the two animal species at adulteration levels as low as 1%. Complementing this, nano-HPLC-ESI-MS/MS provided a comprehensive profile by identifying over 100 bovine-specific peptide markers from β-Lg, α-La, albumin, lactoferrin, and osteopontin. Both methods ensured precise detection and quantification of bovine milk adulteration in complex matrices like pasta filata cheeses, achieving high sensitivity even at minimal adulteration levels. Accordingly, the proposed dual proteomics-based approach overcomes challenges associated with whey protein polymorphism, heat treatment, and processing variability, and complements casein-based methodologies already validated under European standards. This integrated framework of analyses focused on whey and casein fraction enhances the reliability of adulteration detection and safeguards the authenticity of PDO buffalo mozzarella, upholding its unique quality and integrity.

## 1. Introduction

PDO (Protected Designation of Origin) Mozzarella di Bufala Campana (MdBC) is one of Italy’s most rigorously regulated cheeses, renowned for its authenticity and distinctive organoleptic properties. However, adulteration with bovine milk continues to pose a significant challenge for MdBC producers and consumers, since it generates discontent in the costumer expectations and results in economic damage to the businesses at private and national levels. Various speciation methods have been proposed to this purpose that are based on Fourier transform infrared (FTIR) [1] or nuclear magnetic resonance (NMR) [2,3] spectroscopy, 2D electrophoresis [4], capillary electrophoresis [5], high-performance liquid chromatography (HPLC) [6,7], HPLC coupled to electrospray (ESI)−mass spectrometry (MS) [8,9], immunoenzymatic [10,11], MALDI-TOF-MS fingerprinting [12,13], and polymerase chain-reaction-based [14,15,16,17] assays. The above-mentioned approaches sometimes have the disadvantage of having a limited sensitivity in adulteration detection or being complex and time-consuming as a result of multiple experimental steps and long instrument acquisition times.

The official Italian method for detecting this adulteration relies on HPLC measurements to identify β-lactoglobulin A (β-Lg A), a whey protein present in bovine milk but absent in the buffalo counterpart [18]. While this method offers the advantage of rapid detection, it is thought to be without critical limitations, particularly when applied to cheeses subjected to diverse processing conditions. However, one major drawback of this method arises from the low proportion of whey proteins in mozzarella, which account for less than 1% of total cheese content. Minor thermal denaturation or protein losses during cheesemaking can significantly compromise the detectability of bovine β-Lg A. Furthermore, interfering compounds such as Maillard reaction products, formed during curd stretching, can obscure or misidentify bovine β-Lg peaks in authentic mozzarella. These factors not only increase the risk of false negatives, but also highly affect measurement consistency and limit the accurate interpretation of results.

Given these limitations, alternative detection approaches have been developed. The official European method, which focuses on the electrophoretic measurement of the far more abundant casein fraction (constituting over 97% of cheese proteins), has demonstrated a higher reliability in detecting bovine milk contaminations [19]. Recently, MALDI-TOF-MS fingerprinting approaches have also received renewed attention, based on their high specificity in differentiating bovine versus buffalo proteins according to molecular mass values, as well as their speed of execution, although they have shown a detection threshold of approximately 5% *v*/*v* [13,20,21].

In this study, we propose a proteome-inspired strategy based on the analysis of the tryptic digests from the whey fraction of milk samples treated at pH 4.6 or of mozzarella pieces undergoing exhaustive pressing. This method focuses on identifying proteotypic peptide markers to enhance specificity and robustness in distinguishing between bovine and buffalo proteins. By providing a greater accuracy in detecting adulteration, this approach aims to strengthen the safeguarding of the authenticity in PDO MdBC.

## 2. Materials and Methods

### 2.1. Materials

Acetic acid, sodium acetate, and ammonium bicarbonate (AMBIC) were purchased from Carlo Erba (Milan, Italy). Dithiothreitol (DTT) was obtained from Applichem (Darmstadt, Germany). Ultrapure water was prepared using a Milli-Q system (Millipore, Bedford, MA, USA). Iodoacetamide (IAM) and sequencing-grade TPCK-modified trypsin were supplied by Promega (Madison, WI, USA). Buffalo milk was obtained during milking at the Foreste Garofalo buffalo farm (Santa Maria Capua Vetere, Caserta, Italy), while pasteurized buffalo milk (73 °C for 20 s) was sourced from a nearby dairy plant. Mozzarella cheese was produced from this milk on the same day at the same facility. For comparative purposes, sealed samples of PDO MdBC, previously identified using the official Italian analytical method as adulterated with 1.2%, 1.4%, and 1.5% bovine milk, were provided by regulatory authorities. Pasteurized bovine milk was acquired from Parmalat (Caserta, Italy).

### 2.2. Whey and Casein Fractionation

Whey and casein fractions were separated from pasteurized buffalo and bovine milk via isoelectric precipitation at pH 4.6, and three biological replicates of each milk type were used. Calibration curves were prepared using triplicate mixtures of buffalo whey spiked with bovine whey at 1%, 3%, 5%, 10%, 20%, and 30% *v*/*v*.

### 2.3. Sample Preparation and Pressing

Three mozzarella cheese samples underwent exhaustive pressing to remove whey and obtain dewatered mozzarella. The protein concentration of each sample was quantified using a FoodScan™ 2 Lab Pro (FOSS Electric, FOSS Italia, Padova, Italy). The whey samples were further centrifuged using Amicon 10 kDa ultrafiltration columns (Millipore), and the retained protein fraction (devoid of peptides) was reconstituted in 150 µL of 0.4% *w*/*v* AMBIC.

### 2.4. HPLC Separation of Whey Proteins

Chromatographic analysis following the Official Italian Methodology was performed in technical triplicate on the pH 4.6 soluble protein fraction extracted from MdBC cheese samples, pasteurized buffalo milk spiked with variable percentage values of pasteurized bovine milk, and pasteurized bovine milk, used as reference sample. The samples were fractionated by reversed-phase HPLC on HP 1100 modular system (Agilent Technology, Palo Alto, CA, USA) using a reversed-phase Vydac (Hesperia, CA, USA) C4 column (214TP52, 5 μm, 250 *×* 2.1 mm). Fifty μL of the pH 4.6 soluble fraction was injected. Solvents A and B were trifluoracetic acid (1 mL/L) in water and in acetonitrile, respectively. A linear gradient from 20% to 50% was applied over 60 min at a flow rate of 0.2 mL/min. Column effluents were monitored by UV detection at 280 nm.

### 2.5. Reduction, Alkylation, and Tryptic Digestion

Fifteen µL of 100 mM DTT was added to the material from the pH 4.6 soluble protein fraction of mozzarella cheese samples or pasteurized buffalo milk spiked with variable percentage values of pasteurized bovine milk, and the mixture was incubated at 60 °C for 1 h in a thermomixer. Subsequently, 50 µL of 120 mM IAM was added, and the solution was maintained at room temperature in the dark for 1 h. Trypsin digestion was initiated by adding 20 µL of sequencing-grade trypsin solution (1 mg/mL), followed by incubation at 37 °C overnight. Digestion was halted by cooling the samples on ice for 10 min. The resulting solution was dried and reconstituted in 20 µL of 50% *v*/*v* acetonitrile (ACN) containing 0.1% *v*/*v* trifluoroacetic acid (TFA) and finally desalted with ZipTip™ C18 tips.

### 2.6. Mass Spectrometry Analysis

Protein and peptide samples were analyzed by MALDI-TOF-MS using an Ul-traflexExtreme mass spectrometer (Bruker Daltonics, Billerica, MA, USA). Spectral acquisition methods were developed to maximize the number of signals present in the corresponding mass spectra and the corresponding signal-to-noise ratio values.

An aliquot (20 µL) of the reconstituted retained protein fraction was desalted with a ZipTip™ C4 device and directly analyzed by MALDI-TOF-MS for the generation of the corresponding protein profile. Each sample (0.5 μL) was mixed with 0.5 µL of sinapinic acid solution (10 mg/mL in 30% *v*/*v* ACN containing 0.1% *v*/*v* TFA; Bruker Daltonics, Bremen, Germany) and spotted onto a ground steel plate (Bruker Daltonics). Spot samples were air-dried before analysis. Spectra were recorded in the positive linear mode over an *m*/*z* range of 5000–20,000 using FlexControl 3.4 software. The instrument settings included laser frequency, 1000 Hz; ion source 1 voltage, 25.19 kV; ion source 2 voltage, 23.59 kV; lens voltage, 7.50 kV; and sample rate, 0.31. Five independent spectra (1000 shots at random positions on the same target place for spectrum) were automatically collected, calibrated externally by using the Protein Calibration Standard 1 (Bruker Daltonics), and subsequently analyzed.

An aliquot (20 µL) of the protein digest was desalted with a ZipTip™ C18 device and directly analyzed by MALDI-TOF-MS for the generation of the corresponding peptide profile. Each sample (0.5 μL) was mixed with 0.5 μL of a solution of α-cyano-4-hydroxycinnamic acid (25 mg/mL in 30% *v*/*v* ACN containing 0.1% *v*/*v* TFA; Bruker Daltonics, Bremen, Germany), spotted onto the instrument target reported above, and dried at room temperature. The spectra were recorded in the positive reflectron mode over an *m*/*z* range of 500–5000 using FlexControl 3.4 software. The instrument settings included laser frequency, 1000 Hz; ion source 1 voltage, 25.19 kV; ion source 2 voltage, 22.50 kV; lens voltage, 8.50 kV; reflector 1, 26.83 kV; reflector 2, 13.75 kV; and sample rate, 0.63. Five independent spectra (500 shots at random positions on the same target place for spectrum) were automatically collected, calibrated externally by using Peptide Calibration Standard 2 (Bruker Daltonics), and subsequently analyzed.

FlexAnalysis (version 3.4) software packages (Bruker Daltonics) were used for the analysis of all MALDI-TOF-MS data, which included spectral mass adjustment (compression by a factor of 10 in the total mass range), optional smoothing (using the Savitsky−Golay algorithm with a frame size of 25 Da), spectral baseline subtraction, normalization, internal peak alignment, and peak picking.

### 2.7. Nano-HPLC-ESI-Q-Orbitrap-MS/MS

Nano-HPLC-ESI-Q-Orbitrap-MS/MS analysis of the hydrolyzed whey peptide mixtures (100 ng) was performed in technical triplicate using an Ultimate 3000 ultra-high-performance nano-liquid chromatography system (ThermoFisher Scientific, San Jose, CA, USA) coupled to a Q-Exactive Orbitrap Plus mass spectrometer (ThermoFisher Scientific). Peptides were loaded via an autosampler onto an EASY-Spray™ PepMap RP C 18 column (15 cm × 75 µm, 3 µm particle diameter, 100 Å pore size) (ThermoFisher Scientific). Elution was carried out with solvent A (0.1% *v*/*v* formic acid in water) and solvent B (0.1% *v*/*v* formic acid in ACN) at a 300 nL/min flow rate. The column was equilibrated at 0% solvent B for 3 min, followed by a linear gradient of solvent B from 0% to 45% over 60 min. The mass spectrometer operated in data-dependent acquisition mode, with all MS spectra acquired in positive ionization mode over the range of *m*/*z* 350–1600. The top 10 ions in MS were selected for fragmentation in MS/MS mode. Precursor spectra were acquired at a resolving power of 70,000 full width at half maximum (FWHM), with automatic gain control (AGC) targets set to 1 × 10^6^ for full MS and 1 × 10^5^ ions for MS/MS spectra and a maximum ion injection time of 100 ms. MS/MS fragmentation spectra were acquired at a resolving power of 17,500 (FWHM), with a dynamic exclusion window of 10 s. Parent ions with a net charge greater than 6 were excluded from the selection for fragmentation. Data were analyzed using Xcalibur software (v. 2.0).

## 3. Results and Discussion

### 3.1. Stoichiometric Concerns in the Official Italian Method for Adulteration Analysis

A public laboratory recently reported that three samples of MdBC contained 1.2%, 1.4%, and 1.5% bovine milk, respectively, as determined by the official Italian method targeting bovine β-lactoglobulin A (β-Lg A). This method identifies this bovine variant of β-Lg, which generally occurs as two genetic forms, namely A and B, but does not fully capture the complexities of cheesemaking processes. During mozzarella production, most whey is drained, leaving only a small fraction of whey proteins (about 1% of total protein) in the final cheese. Furthermore, near-boiling water added during curd spinning dilutes the whey proteins, reducing their recovery and reliability as markers of adulteration.

To investigate this limitation, we mechanically pressed the above-reported adulterated mozzarella samples to extract whey and determine the protein content of both the whey and the cheese. In 100 g of mozzarella, about 0.64 g consisted of whey proteins in all suspected adulterated MdBC samples, of which 0.33, 0.34, and 0.35 g was recovered in about 55 g of expressed liquid. Assuming that β-LG is about 55% of total whey proteins and that variant A constitutes half of all β-Lg isoforms, the concentration of bovine β-Lg A quantified in the expressed fluids was approximately 0.091 ± 0.009%, 0.094 ± 0.011%, and 0.096 ± 0.012%, respectively. These values contrasted sharply with the 1.2%, 1.4%, and 1.5% bovine milk contamination reported in the application of the official method, underscoring its potential shortcomings when applied to processed cheeses. Although HPLC is a rapid and cost-effective tool, its reliance on bovine β-Lg A as a sole marker poses serious risks for misinterpretation, particularly when calibration relies on raw milk standards or assumes well-defined bovine β-Lg A/B ratios. By contrast, casein markers, which constitute >97% of total mozzarella proteins, are more robust to thermal denaturation and processing variability. Thus, validated methods targeting casein or combining casein and β-Lg A analyses might offer a more reliable approach for quantifying bovine adulteration.

### 3.2. Limitations of the Official Italian Methodology

**Figure 1:**
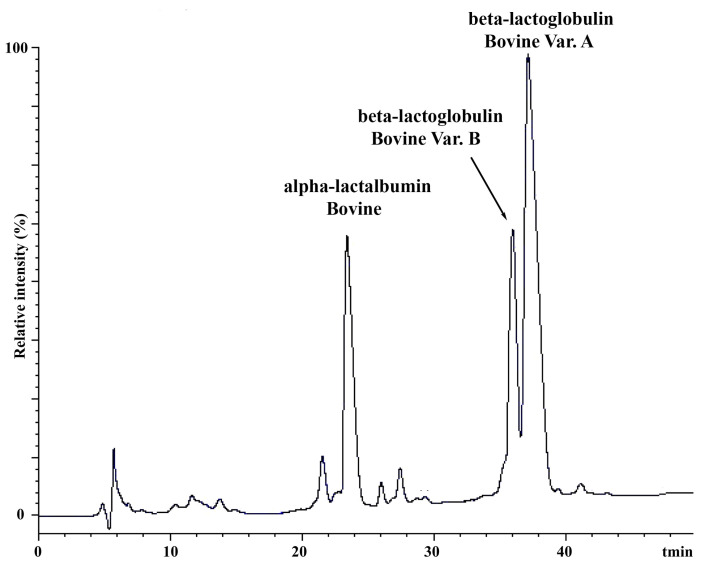
HPLC chromatogram of whey proteins from commercial pasteurized bovine milk showing peaks corresponding to bovine α-lactalbumin A (α-La A), bovine β-lactoglobulin A (β-Lg A), and bovine β-lactoglobulin B (β-Lg B).

To explore scenarios where authentic cheeses might be misclassified as adulterated, we analyzed by HPLC the pH 4.6 soluble proteins from the above-reported MdBC samples classified as containing 1.2%, 1.4%, and 1.5% bovine milk. Under the official Italian protocol, buffalo β-Lg is the only buffalo lactoglobulin species detected during chromatographic analysis, although coeluting with bovine β-Lg B. As reported above, the β-Lg A/β-Lg B ratio can vary naturally in bovine milk, ranging from 0% to 100%. In our analysis of pasteurized bovine milk, about 69 ± 2% was β-Lg A, and nearly 31 ± 1% was β-Lg B (Figure 1). This dairy product was further used to generate adulterated buffalo milk samples with variable percentages of contamination, which were then utilized as reference materials.

We mentioned above that the reliance of the official Italian methodology based on raw milk standards suffers with a certain variability associated with genetic polymorphisms. In this study, we have also considered that heat denaturation of whey proteins during cheese making can significantly reduce the levels of soluble β-Lg at pH 4.6, with respect to those observed when the HPLC instrument is calibrated with raw milk samples, leading to an underestimation of MdBC adulteration. Additionally, the Maillard reaction and the related lactose-associated protein adducts can alter the retention time of the measured analytes in HPLC, complicating comparisons with raw milk standards. Finally, curd stretching in buffalo mozzarella production involves fluctuating temperatures and manual or mechanical techniques, complicating efforts to standardize adulteration models.

**Figure 2:**
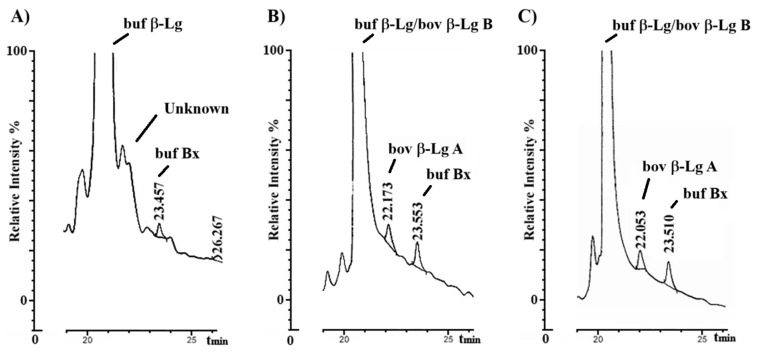
The final segment of HPLC chromatograms obtained using the official Italian methodology applied to the pH 4.6 soluble protein fraction of pasteurized buffalo milk (**A**), pasteurized buffalo milk spiked with 1% *v*/*v* bovine milk (**B**), and the MdBC sample classified as being adulterated with 1.5% *v*/*v* pasteurized bovine milk (**C**). The peak indicated as unknown highlights a compound potentially corresponding to β-Lg A, which was later assigned by proteomics. Peaks related to buffalo β-Lg, bovine β-Lg B, bovine β-Lg A, and buffalo-specific Bx are labeled.

Figure 2 shows the final segment of the HPLC chromatogram resulting from the analysis according to the official Italian methodology of the pH 4.6 soluble protein fractions deriving from pure pasteurized buffalo milk, pasteurized buffalo milk spiked with 1% *v*/*v* pasteurized bovine milk, and the above-reported MdBC sample classified as containing 1.5% bovine milk. According to what was reported in the official method, three peaks corresponding to buffalo β-Lg (coeluting with bovine β-Lg B), bovine β-Lg A, and an unknown buffalo component termed Bx were observed in all samples. As expected, buffalo β-Lg peak co-eluted with bovine β-Lg B. This phenomenon was ascribed to the unique substitution differentiating these proteins at their C-terminus (Ile162 in bovine vs. Val162 in buffalo β-Lg), which did not affect their retention time [22]. Additional peaks eluting after the β-Lg A/B region reflected other buffalo-specific components. Surprisingly, specific peaks with a similar retention time as bovine β-Lg A were observed in the HPLC profile of pure buffalo milk. On the other hand, comparable levels of bovine β-Lg A were measured in the sample of buffalo milk spiked with 1% *v*/*v* bovine counterpart, as well as in the MdBC sample classified as adulterated with 1.5% *v*/*v* bovine milk. Similar findings were observed for the other MdBC samples claimed to have a 1.2% and 1.4% *v*/*v* bovine contamination based on the official Italian method. These results and the proximity in the chromatogram of the analytes essential to assess MdBC adulteration emphasized the need for the use of a complementary analytical technique for definitive compound assignment, such as MALDI-TOF-MS.

### 3.3. MALDI-TOF-MS Analysis of Buffalo β-Lg and Related Protein Components

**Figure 3:**
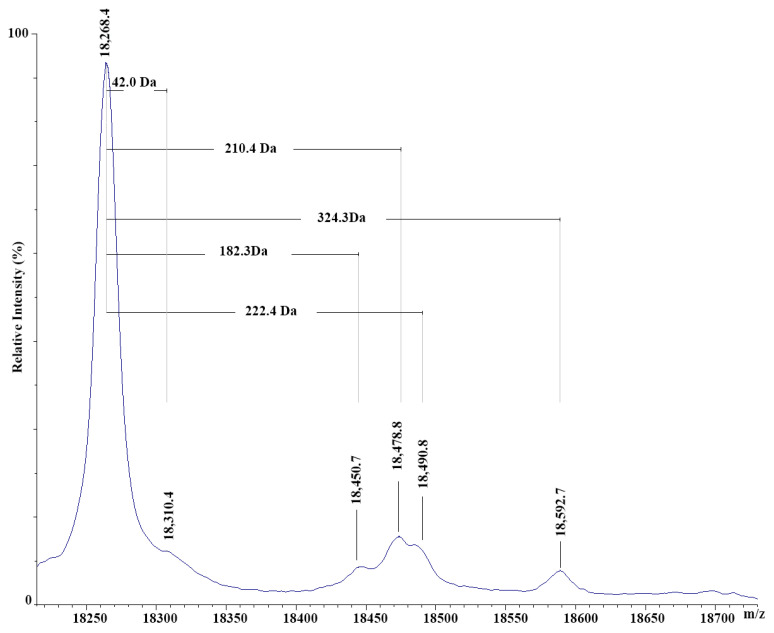
MALDI-TOF mass spectrum of the pH 4.6 whey fraction from pasteurized buffalo milk showing peaks for native buffalo β-lactoglobulin ([M-H]^+^ signal at *m*/*z* 18,268.4) and its N-acetylated ([M-H]^+^ signal at *m*/*z* 18,310.4), N-lauroylated ([M-H]^+^ signal at *m*/*z* 18,450.7), N-myristoylated ([M-H]^+^ signal at *m*/*z* 18,478.8), N-pentadecenoylated ([M-H]^+^ signal at *m*/*z* 18,490.8), and lactosylated ([M-H]^+^ signal at *m*/*z* 18,592.7) derivatives.

To further investigate the nature of whey proteins occurring in buffalo milk, a dedicated MALDI-TOF-MS analysis was accomplished. Figure 3 shows the MALDI-TOF mass spectrum of the β-Lg derivatives occurring in a sample of pasteurized buffalo milk, including those with a mass value exceeding that of the native protein (theor. [M-H]^+^ signal at *m*/*z* 18,267.2; exp. [M-H]^+^ signal at *m*/*z* 18,268.4). This figure well describes the N-lauroylated form of β-Lg that was already associated with peak Bx in the chromatogram of buffalo milk (Figure 2) [23]. Additional components were tentatively assigned to β-Lg N-myristoylated and N-pentadecenoylated derivatives, which are described here for the first time in pasteurized buffalo milk. Another evident [M-H]^+^ signal was associated with the well-known Amadori product of buffalo β-Lg with lactose [24]. Finally, a minor species was tentatively ascribed to the acetylated β-Lg derivative formed via degradation of the above-reported early product of the Maillard reaction [25].

The different β-Lg derivatives observed in the MALDI-TOF mass spectrum of pasteurized buffalo milk were justificative of the complex pattern of minor species recorded in the corresponding HPLC profile (Figure 2A). The different modified β-Lg species bearing similar long-chain fatty acids may coelute within the animal-specific peak Bx described as an invariant marker in the official HPLC method. On the other hand, the more hydrophilic buffalo β-Lg adducts corresponding to the Amadori derivative with lactose and its acetylated degradation product should hypothetically elute in the chromatographic region corresponding to bovine β-Lg A, justifying the peak area changes observed in buffalo milk samples depending on the intensity/duration of their heat treatment and on other processing variables. All of these observations posed a certain concern regarding the possible occurrence of measurement inconsistencies in the official Italian methodology, introducing composition uncertainty in the assessment of adulterated MdBC samples.

### 3.4. MALDI-TOF-MS Analysis of MdBC Samples Classified as Including Bovine Milk

**Figure 4:**
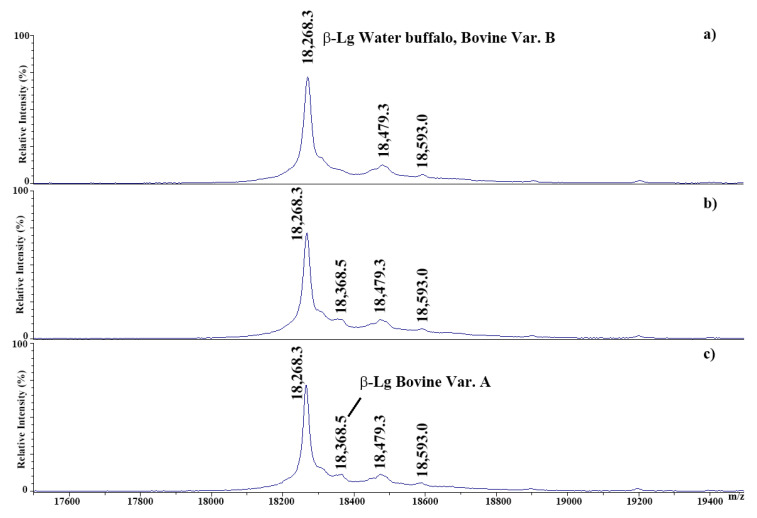
MALDI-TOF mass spectra of the pH 4.6 soluble protein fraction from pasteurized buffalo milk spiked with different amounts of pasteurized bovine milk at 1% (**a**), 3% (**b**), and 5% (**c**) *v*/*v* addiction.

To further investigate the occurrence of bovine β-Lg A in the suspect samples of adulterated MdBC containing 1.2%, 1.4%, and 1.5% bovine milk, as ascertained by the official Italian methodology, a dedicated MALDI-TOF-MS analysis was performed. Despite the fact that the official method assigned a certain level of bovine components in the above-reported adulterated samples, no β-Lg A was detected in all cases. To evaluate the MALDI TOF-MS method’s sensitivity, the pH 4.6 soluble protein fraction from pasteurized buffalo milk spiked with 1%, 3%, or 5% *v*/*v* pasteurized bovine milk was subjected to mass spectrometric analysis (Figure 4).

Distinct signals associated with buffalo β-Lg, bovine β-Lg A, buffalo N-lauroylated β-Lg, and buffalo N-lactosylated β-Lg adduct signals were evident in 3% and 5% *v*/*v* mixtures. Conversely, no signal related to β-Lg A was detectable in the spectrum of buffalo milk having 1% *v*/*v* bovine adulteration, thus highlighting a certain limitation in detecting minimal contamination levels using the MALDI-TOF-MS method alone. These limits were already considered in the development of dedicated methods incorporating casein analysis or proteomics-based multi-marker approaches to enhance the detection sensitivity and quantitation of adulterations with bovine milk in MdBC productions [20,21].

### 3.5. Improving β-Lg Detection Through Trypsinolysis

To overcome the limitations of detecting β-Lg A in its intact protein form, we used a proteomic approach involving MALDI-TOF-MS and nano-HPLC-ESI-MS/MS analysis of the protein tryptic digest of the pH 4.6 whey fraction, significantly enhancing sensitivity and resolution of analysis [26]. In silico digestion with trypsin of bovine β-Lg A predicted a key peptide (61–69) with a [M-H]^+^ signal at *m*/*z* 1121.44, largely differing in mass value with respect to the bovine β-Lg B and buffalo β-Lg peptide counterparts at *m*/*z* 1063.44, due to an Asp → Gly substitution at position 64 (Table 1). Similarly, in silico digestion of bovine β-Lg A predicted another key peptide (102–124) with a [M-H]^+^ signal at *m*/*z* 2672.21, well distinct from the bovine β-Lg B and buffalo β-Lg peptide counterparts at *m*/*z* 2644.18, including Val → Ala substitution at position 118. Both fragments might serve as proteotypic markers, enabling unambiguous identification of bovine β-Lg A in buffalo mozzarella. Additionally, the C-terminal substitution (Val162 → Ile162) distinguished buffalo β-Lg from both bovine variants (β-Lg A and β-Lg B). Specifically, the peptide spanning residues 149–162 in buffalo β-Lg ([M-H]^+^ signal at *m*/*z* 1643.76) might be readily distinguishable from its bovine counterparts ([M-H]^+^ signal at *m*/*z* 1657.78). Thus, the simultaneous detection of signals at *m*/*z* 1121.44 and 2672.21 can confirm the presence of bovine β-Lg A in a sample containing buffalo β-Lg, while the occurrence of peaks at *m*/*z* 1643.76 (buffalo) and 1657.78 (bovine) can indicate that both species are present (Table 1).

**Figure 5:**
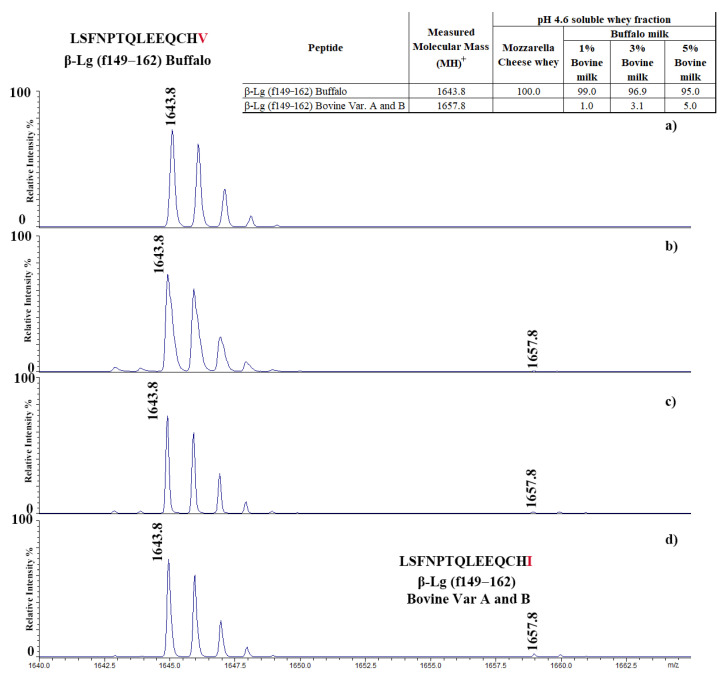
MALDI-TOF mass spectra of the pH 4.6 soluble protein fraction extracted from an adulterated MdBC sample putatively containing 1.5% bovine milk according to the official Italian methodology (**a**), and pasteurized buffalo milk spiked with 1% (**b**), 3% (**c**), and 5% (**d**) *v*/*v* of the bovine counterpart. Samples were subjected to trypsinolysis, as reported in the experimental section, before MALDI-TOF-MS analysis.

These findings underscore the effectiveness of using proteotypic peptides as molecular markers for unequivocally detecting adulteration of buffalo dairy products. We applied this proteomic approach to analyze the pH 4.6 soluble protein fractions extracted from the above-reported adulterated MdBC sample supposed to contain 1.5% cow’s milk (Figure 5a) and pasteurized buffalo whey spiked with 1%, 3%, and 5% *v*/*v* pasteurized bovine whey (Figure 5b–d). The proteotypic peptide (149–162) specific to bovine β-Lg A and B ([M-H]^+^ signal at *m*/*z* 1657.80) was identified in all spiked samples, with detection limits as low as 1%. It is worth mentioning that this peptide was absent from the above-reported adulterated MdBC samples, posing concerns about the consistency of the official Italian methodology. The inset table in Figure 5 quantifies the bovine β-Lg A peptide, underscoring the sensitivity of this method in detecting minimal levels of milk adulteration. These results have highlighted the robustness and precision of MALDI-TOF-MS analysis of tryptic digests in confirming and quantifying bovine milk contaminations in MdBC, offering a reliable tool for detecting bovine adulteration even in complex matrices.

### 3.6. Enhanced Analytical Methodology Using α-La as a Marker

Given the analytical challenges of varying β-Lg A/B ratios in raw bovine milk, α-lactalbumin (α-La) was also evaluated as an alternative adulteration marker to circumvent the above-reported uncertainties due to bovine β-Lg polymorphisms. In the Italian bovine population, α-La isoform B is prevalent, whereas α-La isoform B predominates in the Italian Mediterranean buffalo breed, with the A variant exhibiting an extremely low allele frequency [27]. Consistently, large-scale HPLC analysis of buffalo milk identified only α-La variant B, minimizing the impact of whey protein polymorphism on the robustness of the corresponding calibration curve.

To replicate mozzarella processing, buffalo and bovine milks were pasteurized separately and then mixed to yield buffalo samples containing 1%, 3%, 5%, 10%, 20%, and 30% *v*/*v* bovine counterpart. After adjusting the pH to 4.6, the soluble protein fraction was passed through a 10 kDa cutoff membrane. The retained proteins were divided into two aliquots, as follows: one underwent direct trypsinolysis, while the other was reduced and alkylated before proteolytic digestion. Both samples were subsequently analyzed by MALDI-TOF-MS.

#### 3.6.1. MALDI-TOF-MS and Nano-LC-ESI-MS-MS Calibration Curves for β-Lg and α-La

Based on the expected mass signals of the proteotypic peptides, both before and after alkylation of buffalo and bovine β-Lg and α-La (Table 2), calibration curves for β-Lg detection were developed by plotting the ratio of MALDI-TOF-MS signal intensities of the bovine β-Lg A and B proteotypic peptide (149–162), with respect to the corresponding peptide from buffalo β-Lg.

The resulting equations, derived from different concentration ranges for three biological replicates, were as follows:

For the 1–5% *v*/*v* adulteration range: y = 1.757x + 0.017, R2 = 0.9520 (Figure S1).

For the 10–30% *v*/*v* adulteration range: y = 1.2548x − 0.0997, R2 = 0.9803 (Figure S2).

A similar approach was applied to α-La, using the ratio of MALDI-TOF-MS signal intensities between bovine and buffalo proteotypic peptides. For the 10–30% *v*/*v* adulteration range, focusing on the 42-amino-acid-long peptide (17–58), the resulting equation resulting from the analysis of three biological replicates was as follows: y = 1.3084x + 0.0436y, R^2^ = 0.9954 (Figure S3).

Detection of bovine whey adulteration ranging from 1 to 30% *v*/*v* was readily achieved using of the reduced β-Lg (149–162) peptide. In contrast, α-La (17–58) alone showed insufficient sensitivity to detect adulteration levels below 5% *v*/*v*. However, method sensitivity was significantly improved by carboxymethylating Cys28 in α-La (17–58), which enabled a calibration curve for the 1–5% *v*/*v* adulteration range based on the following three biological replicates: y = 0.2974x + 0.0066, R^2^ = 0.8472 (Figure S4).

These calibration curves underscored the efficacy of the MALDI-TOF-MS method in quantifying bovine milk contamination in the buffalo counterpart. This approach demonstrated notable precision, particularly in the 10–30% adulteration range, by differentiating and quantifying key proteotypic peptides from both β-Lg and α-La. It provided a robust and sensitive means of detecting milk adulteration at the molecular level.

Above-mentioned proteotypic peptides also enabled nano-HPLC-ESI-MS/MS detection of adulteration of pasteurized buffalo milk spiked with varying proportions of the bovine counterpart. The β-Lg proteotypic peptide (149–162) was detected across all adulteration levels, whereas α-La peptide (17–58) was identified only in samples containing at least 5% *v*/*v* bovine milk. To quantify adulteration levels, a calibration curve was generated by plotting the signal ion intensity values of the β-Lg peptides from three biological replicates of buffalo and bovine milk over a 0–30% *v*/*v* adulteration range. The resulting linear regression equations were y = 1.1551x − 1.1341 (R^2^ = 0.9977) for the 0–5% *v*/*v* adulteration range and y = 8.7669x − 0.3459 (R^2^ = 0.9922) for the 10–30% *v*/*v* adulteration range (Figure S5).

Table 3 compares the actual versus measured adulteration percentages using the above-mentioned regression equations, demonstrating the method’s accuracy across a range of adulteration levels from trace amounts (1% *v*/*v*) to major contamination (30% *v*/*v*).

These results confirmed the capability of the mass spectrometry-based proteomic approach to detect adulteration by identifying proteotypic peptides according to their unique sequence and mass value. Unlike the European standard method [19,20], which relies on isoelectric focusing and is subjected to a 0.5% *v*/*v* quantitative margin of error, this proteomic approach provided precise and reliable detection. Under this dedicated procedure, the identification of any bovine-specific peptide indicated adulteration, whether intentional or accidental.

#### 3.6.2. Detection of Bovine Milk Addition in MdBC via Nano-HPLC-ESI-MS/MS Analysis

To evaluate the sensitivity of the above-reported proteomic approach for detecting bovine whey markers, we subjected various samples to tryptic digestion, including the pH 4.6 soluble protein fraction extracted from the following: (i) pure pasteurized buffalo milk; (ii) pure pasteurized bovine milk; (iii) pure pasteurized buffalo milk spiked with the bovine counterpart at 1%, 3%, 5%, 10%, 20%, and 30% *v*/*v*; and (iv) the MdBC sample containing 1.5% bovine milk, according to the official Italian methodology. Nano-LC-ESI-MS/MS analysis revealed that pure buffalo whey contained a total of 305 peptides, whereas pure bovine whey yielded 244 peptides, demonstrating clear proteomic differentiation (Table 4).

For artificial milk mixtures, the number of bovine-specific peptides decreased proportionally with the reduction in bovine milk concentration. Notably, bovine proteotypic peptides (147 in number) were detectable even at 1% adulteration levels, underscoring the method’s high sensitivity.

The data presented in Table S1 highlight peptides specific to bovine milk proteins identified in pure bovine milk and in mixed samples of bovine–buffalo milk. In contrast, the MdBC cheese sample previously flagged by the state laboratory as containing 1.5% *v*/*v* adulteration with bovine milk based on β-Lg A analysis showed only 73 buffalo-specific peptides and no bovine-specific peptides (Table S1), demonstrating the absence of cow material in this sample. Similar results were also observed for the other MdBC samples claimed to have 1.2% and 1.4% *v*/*v* bovine contamination, according to the official Italian method. These findings emphasize the limitations of relying solely on β-Lg A as an HPLC adulteration marker, particularly in thermally processed cheese matrices, often generating false positive results.

Among the most abundant bovine-specific peptides identified in spiked mixtures were those derived from albumin, xanthine dehydrogenase/oxidase, lactotransferrin, lactoperoxidase, polymeric immunoglobulin receptor, and osteopontin. Additionally, peptides originating from the four casein fractions were detected in both buffalo and bovine whey samples, likely generated through the action of endogenous milk enzymes and bacterial proteases. These findings demonstrate the robustness of nano-HPLC-ESI-MS/MS in detecting and quantifying milk adulteration, even at trace levels, while highlighting its superiority over traditional methods that depend on the detection of β-Lg A. By leveraging the identification of both proteotypic and general bovine peptides, this proteomic approach provides a reliable and sensitive tool for verifying the authenticity of MdBC.

## 4. Conclusions

This study highlights significant shortcomings in the official Italian method for detecting bovine milk in PDO Mozzarella di Bufala Campana, particularly in cheeses subjected to diverse thermal treatments. The method’s reliance on minor whey protein markers, such as β-Lg A, often leads to misinterpretation when HPLC peaks reflect heat-induced modifications rather than actual adulteration.

While the current method effectively ensures the authenticity of buffalo ricotta PDO, due to its exclusive reliance on whey proteins, it is less reliable for mozzarella, which consists primarily of caseins and has a minimal proportion of whey proteins. On the other hand, the proteomic approach proposed here based on MALDI-TOF-MS and nano-HPLC-ESI-MS/MS analysis of tryptic digests provides precise, molecular-level identification of adulterant peptides. This dual strategy successfully detected over 100 bovine-specific peptides, enabling confident adulteration measurements at levels as low as 1% *v*/*v* and demonstrating high sensitivity, even in complex pasta filata matrices.

Proteomics conclusively identified adulterant bovine whey proteins in challenging analytical scenarios, outperforming traditional methods. By integrating MALDI-TOF-MS and nano-HPLC-ESI-MS/MS, this advanced approach offers a robust and reliable strategy means to detect, quantify, and authenticate milk components. Implementing these proteomic techniques into standard testing protocols would significantly enhance the reliability, authenticity, and overall quality of PDO buffalo mozzarella production. On the other hand, the need for multiple experimental steps and expensive instruments to execute these proteomic analyses suggest their integration within the framework of official trials involving preventive screening of samples according to casein-centered approaches validated under European standards and then their application for definitive assignment of doubt cases.

## Figures and Tables

**Table 1:** Proteotypic peptides distinguishing bovine and buffalo β-lactoglobulins.

Peptide	Observed [M-H]^+^ Signals		
	Bovine β-Lg		Buffalo β-Lg
	Variant A	Variant B	
61–69	1121.44	1063.44	1063.44
102–124	2672.21	2644.18	2644.18
149–162	1657.78	1657.78	1643.76

**Table 2:** Molecular mass value of key reduced and carbamidomethylated (CysCAM) proteotypic peptides of α-La and β-Lg from bovine and buffalo sources. Reported data refer to [M-H]^+^, [M-2H]^+2^, and [M-3H]^+3^ signals, providing species-specific markers for identifying adulteration of buffalo milk and MdBC cheese. Monoisotopic mass values are also reported.

Protein		Sequence	Peptide	[M-H]^+^ Signal		[M-H]^+2^ Signal		[M-3H]^+3^ Signal	
				Red	CysCAM	Red	CysCAM	Red	CysCAM
α-La	bovine	GYGGVSLPEWVCTTFHTSGY- DTQAIVQNNDSTEYGLFQINNK	17–58	4653.14	4711.14	2327.58	2356.09	1552.05	1571.06
	buffalo	DYGGVSLPEWVCTTFHTSGY- DTQAIVQNNDSTEYGLFQINNK	17–58	4710.16	4768.17	2356.58	2385.09	1571.39	1590.40
β-Lg	bovine	LSFNPTQLEEQCHI	149–162	1657.78	1714.80	822.89	851.40		
	buffalo	LSFNPTQLEEQCHV	149–162	1643.76	1700.78	829.90	858.41		

**Table 3:** Actual vs. measured percentages of bovine milk adulteration of the buffalo counterpart spanning from trace levels (1% *v*/*v*) to major contamination (100% *v*/*v*), resulting from the regression equations derived from nano-HPLC-ESI-MS/MS-based proteomic calibration curves.

	Added Bovine Milk (% *v*/*v*)							
**Actual**	0%	1%	3%	5%	10%	20%	30%	100%
**Measured**	0.00	1.16	2.43	3.42	7.98	18.09	25.52	98.96

**Table 4:** Overview of the peptides identified in of the pH 4.6 soluble protein fraction extracted from pure pasteurized buffalo milk spiked with the bovine counterpart at 1%, 3%, 5%, 10%, 20%, and 30% *v*/*v*, whey from pure pasteurized bovine milk, whey from pure pasteurized buffalo milk, and a MdBC sample classified as adulterated with 1.5% *v*/*v* bovine milk, according to the official Italian methodology. Data describe the number of peptides identified from several proteins. Supplementary Material Table S1 illustrates in detail the proteotypic markers of the bovine species used to detect adulteration of buffalo dairy products. All bovine peptides identified in the MdBC sample, and reported in this table, have an identical sequence, with respect to the buffalo counterparts.

Protein Description	Bovine Adulteration (% *v*/*v*)							Buffalo	Mozzarella Cheese
	1%	3%	5%	10%	20%	30%	100%	100%	
Bovine									
Albumin	36	39	38	38	39	40	36	36	9
Alpha-lactalbumin	8	7	8	7	7	6	8	7	5
Alpha-S1-casein	0	0	0	1	1	1	2	0	0
Alpha-S2-casein	5	5	6	5	5	5	7	5	2
Beta-casein	1	2	2	2	2	3	3	1	1
Beta-lactoglobulin	14	14	15	19	16	19	15	24	16
Butyrophilin subfamily 1 member A1	4	3	2	3	3	3	4	6	4
Cathepsin D	2	2	1	0	0	0	0	0	0
Glycosylation-dependent cell adhesion molecule 1	3	3	2	2	3	3	3	3	2
Immunoglobulin light chain, lambda gene cluster	4	4	6	5	6	6	6	4	0
Kappa-casein	1	2	3	3	3	3	3	1	1
Lactoperoxidase	14	13	15	15	12	12	12	21	1
Lactotransferrin	16	20	20	21	18	23	24	19	9
Milk fat globule EGF factor 8 protein	2	0	1	0	0	0	0	0	0
Osteopontin	1	1	1	1	1	1	0	1	1
Perilipin-2	2	2	3	2	1	1	2	2	2
Plasminogen	0	0	0	0	0	0	2	0	0
Polymeric immunoglobulin receptor	12	14	15	14	14	16	11	15	1
Serotransferrin	10	11	10	13	14	14	12	15	4
Vitamin-D-binding protein	8	8	9	5	5	6	8	8	0
Vitamin-K-dependent protein S	1	1	1	1	1	0	0	1	0
Xanthine dehydrogenase/oxidase	3	2	3	10	7	8	8	16	0
Total bovine peptides	147	153	161	167	158	170	166	185	58
Buffalo									
Alpha-lactalbumin	8	7	8	7	7	6	8	7	5
Alpha-S1-casein	3	3	2	2	3	2	3	3	3
Alpha-S2-casein	5	6	7	5	6	5	2	7	8
Beta-casein	2	3	3	3	3	5	4	3	3
Beta-lactoglobulin	16	16	16	21	17	19	15	26	17
Butyrophilin subfamily 1 member A1	4	3	2	3	3	3	4	6	4
Fatty-acid-binding protein, heart	6	6	6	6	6	6	1	7	1
Glycosylation-dependent cell adhesion molecule 1	5	5	5	5	5	5	5	5	8
Kappa-casein	1	1	1	2	1	2	0	2	3
Lactoferrin	1	2	1	0	2	1	1	1	1
Lactotransferrin	25	27	26	27	24	26	18	28	11
L-lactate dehydrogenase	0	0	0	0	0	1	0	0	0
Milk fat globule EGF factor 8 protein	3	0	2	0	0	0	0	0	0
Osteopontin	5	5	5	4	4	3	4	3	5
Perilipin-2	2	2	2	1	1	1	2	1	2
Serotransferrin	2	1	1	1	2	1	2	3	1
Serum amyloid A protein	1	1	1	1	2	2	2	1	1
Xanthine dehydrogenase/oxidase	4	3	4	10	7	9	7	17	0
Total buffalo peptides	93	91	92	98	93	97	78	120	73
Total bovine and buffalo peptides	240	244	253	265	251	267	244	305	131

## Data Availability

The original contributions presented in the study are included in the article, further inquiries can be directed to the corresponding author.

## Supplementary Materials

The following supporting information can be downloaded at: https://www.mdpi.com/article/10.3390/foods14050822/s1, Table S1: List of primers used in the identification of bacterial and yeast strains and their PCR conditions; Figure S1: MALDI-TOF-MS calibration curve of β-Lg (149–162) in the 1–5% range; Figure S2: MALDI-TOF-MS calibration curve of β-Lg (f149–162) in the 10–30% range; Figure S3: MALDI-TOF-MS calibration curve of α-La (f17–58) in the 10–30% range; Figure S4: MALDI-TOF-MS calibration curve of carboxymethylated α-La (f17–58) in the 1–5% range; Figure S5: Nano-HPLC-ESI-MS/MS calibration curve of β-Lg (f149–162) in the 1–5% and 10–30% range.
