# Supplementary material for: Challenges in Using the Official Italian Method to Detect Bovine Whey Proteins in Protected Designation of Origin Buffalo Mozzarella: A Proteomic Approach to Face Observed Limits
Source: Foods. 2025 Feb 27;14(5):822. doi: 10.3390/foods14050822 (PMC11898797; doi:10.3390/foods14050822)

Challenges in Using the Official Italian Method to Detect Bovine Whey Proteins in Protected Designation of Origin Buffalo Mozzarella: A Proteomic Approach to Face Observed Limits

Federica Della Cerra [1], Mariapia Esposito [1], Simonetta Caira [1,*], Andrea Scaloni [1] and Francesco Addeo [2]

[1] Proteomics, Metabolomics & Mass Spectrometry Laboratory, Institute for the Animal Production System in the Mediterranean Environment, National Research Council, 80055 Portici, Italy; federicadellacerra@cnr.it (F.D.C.); mariaesposito@cnr.it (M.E.); andrea.scaloni@cnr.it (A.S.)
[2] Department of Agriculture, University of Naples "Federico II", 80055 Portici, Italy; doglie42@gmail.com
* Correspondence: simonetta.caira@cnr.it; Tel.: +39-392-6081165

Supplementary Material

Supplementary Material Table S1.

Total peptides identified by nano-HPLC-ESI-MS/MS in the pH 4.6 soluble protein fraction extracted from pure pasteurized buffalo milk spiked with the bovine counterpart at 1%, 3%, 5%, 10%, 20%, and 30% v/v, whey from pure pasteurized bovine milk, whey from pure pasteurized buffalo milk, and a MdBC sample classified as adulterated with 1.5% v/v bovine milk according to the official Italian methodology. The latter sample exhibited 58 buffalo peptides and no bovine-specific peptides. Proteotypic sequences associated with buffalo- and bovine-specific peptides are highlighted in bold. A brown color gradation, from lightest to darkest, represents increasing signal intensity values.

Protein description	Start	End	Sequence	LFQ Intensity								
				1%	0.03	0.05	0.1	0.2	0.3	100% BOV	100% BUF	Mozzarella cheese sample
Albumin P02769 · ALBU_BOVIN; XP_006047011.1_Bubalus bubalis	35	44	FKDLGEEHFK	8,75E+06	6,92E+06	0,00E+00	0,00E+00	6,28E+06	0,00E+00	1,12E+07	0,00E+00	0,00E+00
Albumin P02769 · ALBU_BOVIN; XP_006047011.1_Bubalus bubalis	37	44	DLGEEHFK	7,49E+08	1,54E+09	9,06E+08	1,09E+09	1,30E+09	7,13E+08	8,06E+08	1,26E+09	0,00E+00
Albumin P02769 · ALBU_BOVIN	45	65	**GLVLIAFSQYLQQCPFDEHVK**	0,00E+00	0,00E+00	0,00E+00	0,00E+00	0,00E+00	0,00E+00	9,92E+05	0,00E+00	0,00E+00
Albumin P02769 · ALBU_BOVIN; XP_006047011.1_Bubalus bubalis	66	75	LVNELTEFAK	2,63E+09	2,68E+09	3,16E+09	2,09E+09	1,98E+09	1,92E+09	1,61E+09	2,71E+09	1,08E+08
Albumin P02769 · ALBU_BOVIN	76	88	**TCVADESHAGCEK**	1,54E+06	2,88E+06	5,04E+06	2,01E+07	4,69E+07	4,80E+07	6,99E+07	0,00E+00	0,00E+00
Albumin P02769 · ALBU_BOVIN; XP_006047011.1_Bubalus bubalis	89	100	**SLHTLFGDELCK**	2,78E+08	3,49E+08	3,32E+08	3,53E+08	4,98E+08	6,43E+08	2,76E+09	4,29E+08	0,00E+00
Albumin P02769 · ALBU_BOVIN; XP_006047011.1_Bubalus bubalis	106	117	ETYGDMADCCEK	4,61E+07	6,15E+07	3,16E+07	5,06E+07	5,41E+07	5,83E+07	4,75E+07	6,22E+07	0,00E+00
Albumin P02769 · ALBU_BOVIN	123	130	**NECFLSHK**	7,71E+06	3,19E+07	3,04E+07	8,23E+07	1,61E+08	2,25E+08	6,17E+08	0,00E+00	0,00E+00
Albumin P02769 · ALBU_BOVIN; XP_006047011.1_Bubalus bubalis	131	138	DDSPDLPK	7,18E+07	6,78E+07	3,31E+07	6,92E+07	8,30E+07	8,56E+07	2,25E+08	8,73E+07	0,00E+00
Albumin P02769 · ALBU_BOVIN	139	151	**LKPDPNTLCDEFK**	8,57E+07	1,13E+08	1,52E+08	7,40E+07	7,48E+07	8,60E+07	1,68E+08	0,00E+00	0,00E+00
Albumin P02769 · ALBU_BOVIN; XP_006047011.1_Bubalus bubalis	161	167	YLYEIAR	5,86E+09	5,83E+09	3,88E+09	4,49E+09	5,09E+09	4,49E+09	2,75E+09	5,12E+09	1,10E+08

Albumin P02769 · ALBU_BOVIN; XP_006047011.1_Bubalus bubalis	168	183	RHPYFYAPELLYYANK	0,00E+00	0,00E+00	0,00E+00	0,00E+00	0,00E+00	2,01E+06	0,00E+00	0,00E+00	0,00E+00
Albumin P02769 · ALBU_BOVIN; XP_006047011.1_Bubalus bubalis	169	183	HPYFYAPELLYYANK	3,88E+06	2,53E+07	3,81E+07	7,61E+06	7,91E+07	1,41E+07	6,52E+06	4,08E+07	0,00E+00
Albumin P02769 · ALBU_BOVIN; XP_006047011.1_Bubalus bubalis	184	197	YNGVFQECCQAEDK	6,80E+08	7,56E+08	6,67E+08	7,09E+08	5,89E+08	5,68E+08	3,75E+08	7,07E+08	0,00E+00
Albumin P02769 · ALBU_BOVIN; XP_006047011.1_Bubalus bubalis	246	256	FPKAEFVEVTK	0,00E+00	0,00E+00	0,00E+00	0,00E+00	0,00E+00	0,00E+00	0,00E+00	8,41E+05	0,00E+00
Albumin P02769 · ALBU_BOVIN; XP_006047011.1_Bubalus bubalis	249	256	AEFVEVTK	3,00E+09	3,20E+09	2,48E+09	2,70E+09	2,88E+09	2,56E+09	2,22E+09	2,96E+09	0,00E+00
Albumin P02769 · ALBU_BOVIN; XP_006047011.1_Bubalus bubalis	264	280	VHKECCHGDLLECADDR	1,64E+06	3,22E+06	3,72E+05	1,23E+06	7,02E+06	7,44E+06	0,00E+00	3,87E+06	0,00E+00
Albumin P02769 · ALBU_BOVIN; XP_006047011.1_Bubalus bubalis	267	280	ECCHGDLLECADDR	2,78E+09	2,96E+09	2,53E+09	2,93E+09	2,84E+09	2,61E+09	1,56E+09	2,65E+09	0,00E+00
Albumin P02769 · ALBU_BOVIN; XP_006047011.1_Bubalus bubalis	286	297	YICDNQDTISSK	3,79E+09	4,31E+09	3,51E+09	3,81E+09	4,11E+09	3,93E+09	2,26E+09	3,35E+09	1,76E+06
Albumin P02769 · ALBU_BOVIN; XP_006047011.1_Bubalus bubalis	298	309	LKECCDKPLLEK	5,05E+06	5,81E+06	5,15E+07	3,95E+07	1,60E+07	2,10E+07	0,00E+00	4,19E+07	0,00E+00
Albumin P02769 · ALBU_BOVIN; XP_006047011.1_Bubalus bubalis	300	309	ECCDKPLLEK	1,66E+09	1,72E+09	1,20E+09	1,73E+09	1,78E+09	1,42E+09	1,47E+09	1,87E+09	0,00E+00
Albumin P02769 · ALBU_BOVIN	310	318	**SHCIAEVEK**	0,00E+00	9,44E+06	8,84E+06	2,83E+07	2,11E+07	8,36E+07	4,92E+08	0,00E+00	0,00E+00
Albumin P02769 · ALBU_BOVIN; XP_006047011.1_Bubalus bubalis	347	359	DAFLGSFLYEYSR	0,00E+00	1,10E+07	8,00E+06	0,00E+00	0,00E+00	1,49E+07	0,00E+00	1,90E+05	0,00E+00
Albumin P02769 · ALBU_BOVIN; XP_006047011.1_Bubalus bubalis	360	371	RHPEYAVSVLLR	5,01E+08	6,03E+08	1,15E+09	8,43E+08	6,38E+08	7,32E+08	4,20E+08	9,70E+08	9,14E+07
Albumin P02769 · ALBU_BOVIN; XP_006047011.1_Bubalus bubalis	361	371	HPEYAVSVLLR	1,48E+09	1,78E+09	1,44E+09	1,55E+09	1,51E+09	1,85E+09	1,08E+09	1,49E+09	0,00E+00
Albumin P02769 · ALBU_BOVIN; XP_006047011.1_Bubalus bubalis	375	386	EYEATLEECCAK	8,01E+08	9,79E+08	7,88E+08	8,63E+08	8,23E+08	8,44E+08	7,21E+08	8,35E+08	0,00E+00

Albumin P02769 · ALBU_BOVIN; XP_006047011.1_Bubalus bubalis	387	399	DDPHACYSTVFDK	2,79E+09	2,65E+09	2,78E+09	2,50E+09	2,44E+09	1,98E+09	1,60E+09	2,40E+09	0,00E+00
Albumin P02769 · ALBU_BOVIN; XP_006047011.1_Bubalus bubalis	402	412	HLVDEPQNLIK	5,71E+09	5,66E+09	5,91E+09	6,33E+09	5,11E+09	5,05E+09	3,38E+09	5,90E+09	1,47E+08
Albumin P02769 · ALBU_BOVIN; XP_006047011.1_Bubalus bubalis	413	420	QNCDQFEK	1,31E+07	1,48E+07	7,22E+06	1,61E+07	1,79E+07	1,45E+07	2,14E+07	2,25E+07	0,00E+00
Albumin P02769 · ALBU_BOVIN; XP_006047011.1_Bubalus bubalis	421	433	LGEYGFQNALIVR	1,75E+08	2,29E+08	3,44E+08	1,73E+08	2,00E+08	2,03E+08	2,82E+08	1,76E+08	0,00E+00
Albumin P02769 · ALBU_BOVIN; XP_006047011.1_Bubalus bubalis	437	451	KVPQVSTPTLVEVSR	3,13E+09	3,03E+09	2,92E+09	2,47E+09	6,66E+08	2,28E+09	2,18E+09	2,43E+09	1,79E+08
Albumin P02769 · ALBU_BOVIN; XP_006047011.1_Bubalus bubalis	438	451	VPQVSTPTLVEVSR	1,03E+09	1,01E+09	7,09E+08	8,15E+08	7,33E+08	6,89E+08	4,31E+08	8,49E+08	0,00E+00
Albumin P02769 · ALBU_BOVIN; XP_006047011.1_Bubalus bubalis	469	482	MPCTEDYLSLILNR	1,07E+08	1,11E+08	6,71E+07	6,57E+07	5,80E+07	7,18E+07	4,98E+06	9,72E+07	0,00E+00
Albumin P02769 · ALBU_BOVIN; XP_006047011.1_Bubalus bubalis	483	489	LCVLHEK	1,39E+09	1,38E+09	1,08E+09	1,37E+09	1,44E+09	1,14E+09	6,54E+08	1,42E+09	0,00E+00
Albumin P02769 · ALBU_BOVIN; XP_006047011.1_Bubalus bubalis	499	507	CCTESLVNR	2,13E+08	2,31E+08	1,75E+08	8,87E+07	7,28E+07	1,45E+08	5,43E+07	2,50E+08	0,00E+00
Albumin P02769 · ALBU_BOVIN; XP_006047011.1_Bubalus bubalis	508	523	RPCFSALTPDETYVPK	3,82E+08	5,52E+08	6,46E+08	5,13E+08	4,77E+08	3,03E+08	1,63E+08	2,45E+08	0,00E+00
Albumin P02769 · ALBU_BOVIN; XP_006047011.1_Bubalus bubalis	529	544	LFTFHADICTLPDTEK	1,11E+08	1,92E+08	1,48E+08	1,53E+08	1,81E+08	2,03E+08	2,64E+07	3,22E+08	3,32E+06
Albumin P02769 · ALBU_BOVIN; XP_006047011.1_Bubalus bubalis	548	557	KQTALVELLK	5,06E+07	5,80E+07	1,59E+08	6,82E+07	6,40E+07	8,14E+07	4,30E+07	6,27E+07	0,00E+00
Albumin P02769 · ALBU_BOVIN; XP_006047011.1_Bubalus bubalis	549	557	QTALVELLK	2,77E+08	2,43E+08	2,47E+08	2,49E+08	2,08E+08	2,28E+08	1,22E+08	2,31E+08	1,11E+07
Albumin P02769 · ALBU_BOVIN; XP_006047011.1_Bubalus bubalis	562	568	ATEEQLK	0,00E+00	0,00E+00	0,00E+00	4,75E+06	7,80E+06	1,04E+07	0,00E+00	1,12E+07	0,00E+00

Albumin P02769 · ALBU_BOVIN; XP_006047011.1_Bubalus bubalis	569	580	TVMENFVAFVDK	3,53E+08	3,40E+08	3,32E+08	3,10E+08	3,72E+08	2,67E+08	2,03E+08	2,38E+08	2,38E+06
Albumin P02769 · ALBU_BOVIN; XP_006047011.1_Bubalus bubalis	588	597	EACFAVEGPK	0,00E+00	3,75E+07	1,96E+07	4,01E+07	2,14E+08	3,19E+08	1,30E+09	0,00E+00	0,00E+00
Albumin P02769 · ALBU_BOVIN	598	607	**LVVSTQTALA**	4,88E+06	5,11E+06	1,99E+07	3,03E+07	9,72E+07	1,49E+08	7,01E+08	0,00E+00	0,00E+00
Alpha-lactalbumin (P00711 · LALBA_BOVIN; A0A7T0M8A3_BUBBU)	59	79	IWCKDDQNPHSSNICNISCDK	0,00E+00	6,04E+06	8,07E+07	3,65E+07	1,10E+07	0,00E+00	1,84E+07	3,59E+07	0,00E+00
Alpha-lactalbumin (P00711 · LALBA_BOVIN; A0A7T0M8A3_BUBBU)	63	79	DDQNPHSSNICNISCDK	2,97E+10	3,30E+10	3,84E+10	2,61E+10	2,74E+10	2,55E+10	1,94E+10	2,59E+10	3,80E+07
Alpha-lactalbumin (P00711 · LALBA_BOVIN; A0A7T0M8A3_BUBBU)	80	93	FLDDDLTDDIMCVK	6,68E+09	7,58E+09	5,80E+09	6,25E+09	6,96E+09	6,45E+09	2,91E+09	5,69E+09	2,92E+07
Alpha-lactalbumin (P00711 · LALBA_BOVIN; A0A7T0M8A3_BUBBU)	80	94	FLDDDLTDDIMCVKK	0,00E+00	0,00E+00	1,26E+06	0,00E+00	0,00E+00	0,00E+00	0,00E+00	0,00E+00	0,00E+00
Alpha-lactalbumin (P00711 · LALBA_BOVIN; A0A7T0M8A3_BUBBU)	94	108	KILDKVGINYWLAHK	4,58E+06	0,00E+00	0,00E+00	0,00E+00	0,00E+00	1,63E+07	0,00E+00	0,00E+00	0,00E+00
Alpha-lactalbumin (P00711 · LALBA_BOVIN; A0A7T0M8A3_BUBBU)	95	108	ILDKVGINYWLAHK	1,59E+07	1,25E+07	2,46E+07	4,56E+06	5,69E+06	9,16E+06	4,30E+07	2,78E+07	8,14E+06
Alpha-lactalbumin (P00711 · LALBA_BOVIN; A0A7T0M8A3_BUBBU)	99	108	VGINYWLAHK	6,78E+10	7,05E+10	8,37E+10	6,21E+10	5,98E+10	6,53E+10	5,11E+10	5,18E+10	8,23E+10
Alpha-lactalbumin (P00711 · LALBA_BOVIN; A0A7T0M8A3_BUBBU)	109	122	ALCSEKLDQWLCEK	8,91E+06	1,35E+07	4,41E+06	8,77E+06	7,06E+06	0,00E+00	1,05E+07	3,20E+07	0,00E+00
Alpha-lactalbumin (P00711 · LALBA_BOVIN; A0A7T0M8A3_BUBBU)	109	123	ALCSEKLDQWLCEKL	4,52E+06	0,00E+00	0,00E+00	0,00E+00	0,00E+00	0,00E+00	0,00E+00	0,00E+00	0,00E+00
Alpha-lactalbumin (P00711 · LALBA_BOVIN; A0A7T0M8A3_BUBBU)	115	122	LDQWLCEK	7,05E+10	7,70E+10	7,42E+10	6,57E+10	6,81E+10	6,25E+10	5,22E+10	5,28E+10	2,89E+08
Alpha-lactalbumin (P00711 · LALBA_BOVIN; A0A7T0M8A3_BUBBU)	115	123	LDQWLCEKL	0,00E+00	0,00E+00	0,00E+00	0,00E+00	0,00E+00	0,00E+00	3,13E+06	0,00E+00	0,00E+00
Alpha-S1-casein (P02662 · CASA1_BOVIN)	8	22	**HQGLPQEVLNENLLR**	0,00E+00	0,00E+00	0,00E+00	8,03E+05	3,82E+06	2,85E+07	1,01E+09	0,00E+00	0,00E+00

Alpha-S1-casein (P02662 · CASA1_BOVIN)	35	42	EKVNELSK	0,00E+00	0,00E+00	0,00E+00	0,00E+00	0,00E+00	0,00E+00	0,00E+00	0,00E+00	0,00E+00
Alpha-S1-casein (P02662 · CASA1_BOVIN)	43	58	**DIGSESTEDQAMEDIK**	0,00E+00	0,00E+00	0,00E+00	0,00E+00	0,00E+00	0,00E+00	2,25E+06	0,00E+00	0,00E+00
Alpha-S1-casein (Q4F6X6 · Q4F6X6_BUBBU)	23	34	FFVAPFPEVFGK	4,68E+06	2,96E+06	6,67E+06	0,00E+00	1,07E+06	0,00E+00	1,55E+07	5,40E+05	1,91E+09
Alpha-S1-casein (Q4F6X6 · Q4F6X6_BUBBU)	8	22	**HQGLPQGVLNENLLR**	9,09E+06	2,66E+06	2,02E+07	4,54E+08	4,07E+08	4,11E+08	0,00E+00	4,89E+08	6,14E+09
Alpha-S1-casein (Q4F6X6 · Q4F6X6_BUBBU)	91	100	YLGYLEQLLR	3,46E+06	2,26E+06	0,00E+00	4,88E+06	1,56E+07	2,18E+07	2,74E+07	1,94E+06	2,30E+08
Alpha-S1-casein (Q4F6X6 · Q4F6X6_BUBBU)	125	132	EGIHAQQK	0,00E+00	0,00E+00	0,00E+00	0,00E+00	0,00E+00	0,00E+00	3,83E+06	0,00E+00	0,00E+00
Alpha-S2-casein (P02663 · CASA2_BOVIN)	2	21	NTMEHVSSSEESIISQETYK	0,00E+00	0,00E+00	0,00E+00	0,00E+00	0,00E+00	0,00E+00	1,18E+07	0,00E+00	0,00E+00
Alpha-S2-casein (P02663 · CASA2_BOVIN)	25	32	NMAINPSK	0,00E+00	0,00E+00	0,00E+00	0,00E+00	0,00E+00	0,00E+00	3,94E+07	0,00E+00	0,00E+00
Alpha-S2-casein (P02663 · CASA2_BOVIN)	174	181	FALPQYLK	6,66E+06	1,56E+07	9,02E+06	6,23E+07	1,81E+08	2,89E+08	2,57E+09	0,00E+00	0,00E+00
Alpha-S2-casein (P02663 · CASA2_BOVIN)	182	197	TVYQHQKAMKPWIQPK	0,00E+00	0,00E+00	0,00E+00	0,00E+00	0,00E+00	0,00E+00	0,00E+00	3,89E+05	0,00E+00
Alpha-S2-casein (P02663 · CASA2_BOVIN)	189	197	AMKPWIQPK	0,00E+00	0,00E+00	0,00E+00	4,18E+06	1,20E+07	2,26E+07	1,46E+08	0,00E+00	0,00E+00
Alpha-S2-casein (P02663 · CASA2_BOVIN)	138	149	TVDMESTEVFTK	3,27E+06	5,87E+06	2,70E+06	9,44E+06	1,69E+07	3,36E+07	1,19E+08	1,51E+06	0,00E+00
Alpha-S2-casein (O62825_BUBBU)	40	47	NMAIHPSK	0,00E+00	0,00E+00	0,00E+00	0,00E+00	0,00E+00	0,00E+00	0,00E+00	0,00E+00	5,78E+06
Alpha-S2-casein (O62825_BUBBU)	153	164	TVDMESTEVITK	4,60E+07	4,02E+07	3,61E+07	2,85E+07	2,53E+07	2,41E+07	0,00E+00	2,93E+07	7,41E+07
Alpha-S2-casein (O62825_BUBBU)	177	184	FTWPQYLK	0,00E+00	0,00E+00	0,00E+00	0,00E+00	3,06E+06	0,00E+00	0,00E+00	4,62E+06	1,31E+09
Alpha-S2-casein (O62825_BUBBU)	180	187	FAWPQYLK	0,00E+00	0,00E+00	0,00E+00	0,00E+00	0,00E+00	0,00E+00	0,00E+00	1,22E+06	4,78E+08
Alpha-S2-casein (O62825_BUBBU)	188	194	TVYQYQK	0,00E+00	1,65E+07	1,53E+07	7,72E+06	1,03E+07	2,27E+07	0,00E+00	0,00E+00	4,20E+08
Alpha-S2-casein (O62825_BUBBU)	195	203	AMKPWTQPK	2,78E+06	2,18E+06	1,48E+06	1,63E+07	1,51E+07	9,67E+06	0,00E+00	1,75E+07	4,79E+08
Alpha-S2-casein (P02663 · CASA2_BOVIN;B6VPY3_BUBBU)	33	41	ENLCSTFCK	1,63E+07	1,89E+07	3,80E+07	2,70E+07	1,19E+07	3,06E+07	7,89E+07	1,43E+07	0,00E+00
Alpha-S2-casein (P02663 · CASA2_BOVIN;B6VPY3_BUBBU)	81	91	ALNEINQFYQK	0,00E+00	0,00E+00	0,00E+00	0,00E+00	0,00E+00	0,00E+00	0,00E+00	0,00E+00	3,84E+08

Alpha-S2-casein (P02663 · CASA2_BOVIN;B6VPY3_BUBBU)	114	125	RNAVPITPTLNR	3,91E+06	3,32E+06	6,42E+06	0,00E+00	0,00E+00	0,00E+00	0,00E+00	0,00E+00	0,00E+00
Alpha-S2-casein (P02663 · CASA2_BOVIN;B6VPY3_BUBBU)	115	125	NAVPITPTLNR	3,72E+09	3,61E+09	4,01E+09	2,09E+09	2,16E+09	2,59E+09	8,01E+09	1,46E+09	6,32E+09
Alpha-S2-casein (P02663 · CASA2_BOVIN;B6VPY3_BUBBU)	126	136	EQLSTSEENSK	0,00E+00	0,00E+00	2,77E+05	0,00E+00	0,00E+00	0,00E+00	0,00E+00	9,64E+05	0,00E+00
Beta-casein (B7VGH4_BUBBU)	33	48	**FQSEEQQQMEDELQDK**	3,75E+07	6,32E+07	3,28E+07	2,16E+06	0,00E+00	7,57E+06	0,00E+00	1,53E+06	1,25E+09
Beta-casein (B7VGH4_BUBBU)	49	69	**IHPFAQTQSLVYPFPGPIPK**	0,00E+00	1,45E+07	1,58E+08	1,61E+08	1,86E+08	1,33E+08	0,00E+00	2,47E+08	1,71E+10
Beta-casein (A0N0S5_BUBBU)	54	68	**QTQSLVYPFPGPIPK**	0,00E+00	0,00E+00	0,00E+00	1,00E+06	1,10E+06	1,04E+06	0,00E+00	3,25E+06	1,04E+08
Beta-casein (B7VGH4_BUBBU)	69	97	SLPQNIPPLTQTPVVVPPFLQPEIMGVSK	9,12E+07	1,34E+08	7,20E+07	7,59E+07	1,79E+08	6,58E+07	0,00E+00	4,88E+08	1,45E+10
Beta-casein (A0A873X5B1_BUBBU)	114	82	YPVEPFTESQS	0,00E+00	0,00E+00	0,00E+00	0,00E+00	0,00E+00	6,97E+06	1,78E+07	1,00E+07	7,08E+07
Beta-casein (P02666 · CASB_BOVIN; A0A6B9MI77_BUBBU)	33	48	**FQSEEQQQTEDELQDK**	0,00E+00	5,22E+06	8,61E+06	1,83E+07	3,80E+07	9,11E+07	8,73E+08	0,00E+00	0,00E+00
Beta-casein (P02666 · CASB_BOVIN; A0A6B9MI77_BUBBU)	170	183	VLPVPQKAVPYPQR	0,00E+00	0,00E+00	0,00E+00	0,00E+00	0,00E+00	5,00E+06	0,00E+00	0,00E+00	0,00E+00
Beta-casein (P02666 · CASB_BOVIN; A0A6B9MI77_BUBBU)	177	183	AVPYPQR	1,13E+09	1,36E+09	1,36E+09	8,20E+09	9,32E+09	9,01E+09	1,14E+10	7,81E+09	2,20E+10
Beta-casein (P02666 · CASB_BOVIN; A0A6B9MI77_BUBBU)	203	209	GPFPIIV	0,00E+00	0,00E+00	0,00E+00	0,00E+00	0,00E+00	0,00E+00	1,29E+08	0,00E+00	0,00E+00
Beta-lactoglobulin (P02754 · LACB_BOVIN)	1	8	LIVTQTMK	6,73E+10	6,75E+10	6,67E+10	6,63E+10	7,81E+10	7,22E+10	6,05E+10	5,67E+10	2,14E+11
Beta-lactoglobulin (P02754 · LACB_BOVIN)	1	14	LIVTQTMKGLDIQK	0,00E+00	0,00E+00	0,00E+00	0,00E+00	0,00E+00	0,00E+00	1,75E+07	0,00E+00	8,94E+06
Beta-lactoglobulin (P02754 · LACB_BOVIN)	1	40	LIVTQTMKGLDIQKVAGTWYSLAMAASDISLLDAQSAPLR	0,00E+00	0,00E+00	0,00E+00	0,00E+00	0,00E+00	0,00E+00	0,00E+00	6,49E+05	0,00E+00
Beta-lactoglobulin (P02754 · LACB_BOVIN)	77	85	**WENDECAQK**	2,63E+08	4,25E+08	2,09E+08	3,82E+08	5,85E+08	1,22E+09	8,55E+09	0,00E+00	0,00E+00
Beta-lactoglobulin (P02754 · LACB_BOVIN)	77	86	**WENDECAQKK**	0,00E+00	0,00E+00	0,00E+00	0,00E+00	0,00E+00	1,55E+06	5,66E+06	0,00E+00	0,00E+00
Beta-lactoglobulin (P02754 · LACB_BOVIN;)	92	100	**LSFNPTQLEEQCHI**	3,71E+08	1,34E+09	1,88E+09	3,99E+09	7,63E+09	1,28E+10	5,36E+10	0,00E+00	0,00E+00

Beta-lactoglobulin (Q8MHZ7_BUBBU)	4	12	VLVLNTDYK	2,00E+07	9,07E+06	1,04E+07	7,89E+06	2,18E+06	9,80E+06	4,03E+06	6,37E+06	5,24E+06
Beta-lactoglobulin (E9N3M1_BUBBU)	33	39	VAGTWYS	4,34E+07	4,50E+07	0,00E+00	3,46E+07	0,00E+00	0,00E+00	3,08E+07	2,19E+07	0,00E+00
Beta-lactoglobulin (H9CH53_BUBBU)	94	100	VLDTDYK	7,48E+07	3,74E+08	1,64E+08	3,03E+08	1,57E+08	4,44E+08	4,51E+08	1,36E+08	1,37E+09
Beta-lactoglobulin (H9CH53_BUBBU)	94	101	VLDTDYKK	3,95E+07	3,64E+07	8,20E+07	2,15E+08	2,48E+08	1,48E+08	2,25E+07	1,14E+08	3,20E+07
Beta-lactoglobulin (A0A7M1I6E3_BUBBU)	167	180	**LSFNPTQLEEQCHV**	3,23E+10	5,36E+10	5,40E+10	4,57E+10	3,36E+10	3,74E+10	0,00E+00	2,44E+10	9,71E+07
Beta-lactoglobulin (P02754 · LACB_BOVIN; A0A7M1I6E3_BUBBU)	9	40	GLDIQKVAGTWYSLAMAASDISLLDAQSAPLR	0,00E+00	0,00E+00	0,00E+00	0,00E+00	0,00E+00	2,53E+06	4,15E+06	9,10E+06	0,00E+00
Beta-lactoglobulin (P02754 · LACB_BOVIN; A0A7M1I6E3_BUBBU)	15	40	VAGTWYSLAMAASDISLLDAQSAPLR	0,00E+00	0,00E+00	0,00E+00	1,24E+06	0,00E+00	0,00E+00	0,00E+00	8,37E+06	1,17E+06
Beta-lactoglobulin (P02754 · LACB_BOVIN; A0A7M1I6E3_BUBBU)	41	60	VYVEELKPTPEGDLEILLQK	2,05E+10	2,33E+10	1,57E+09	1,04E+10	2,24E+10	1,27E+10	7,26E+09	1,38E+10	3,39E+09
Beta-lactoglobulin (P02754 · LACB_BOVIN; A0A7M1I6E3_BUBBU)	41	69	VYVEELKPTPEGDLEILLQKWENGECAQK	0,00E+00	0,00E+00	0,00E+00	0,00E+00	0,00E+00	0,00E+00	0,00E+00	6,24E+05	0,00E+00
Beta-lactoglobulin (P02754 · LACB_BOVIN; A0A7M1I6E3_BUBBU)	61	69	WENGECAQK	9,20E+09	1,14E+10	6,12E+09	9,29E+09	1,06E+10	9,20E+09	6,74E+09	1,23E+10	0,00E+00
Beta-lactoglobulin (P02754 · LACB_BOVIN; A0A7M1I6E3_BUBBU)	61	70	WENGECAQKK	1,81E+07	1,56E+07	1,36E+07	4,82E+06	1,48E+06	6,49E+06	0,00E+00	5,16E+06	0,00E+00
Beta-lactoglobulin (P02754 · LACB_BOVIN; A0A7M1I6E3_BUBBU)	61	75	WENGECAQKKIIAEK	0,00E+00	0,00E+00	0,00E+00	1,28E+06	1,50E+06	0,00E+00	0,00E+00	0,00E+00	0,00E+00
Beta-lactoglobulin (P02754 · LACB_BOVIN; A0A7M1I6E3_BUBBU)	76	83	TKIPAVFK	1,28E+08	1,27E+08	2,57E+08	5,12E+08	5,87E+08	6,20E+08	9,76E+07	5,49E+08	5,50E+09
Beta-lactoglobulin (P02754 · LACB_BOVIN; A0A7M1I6E3_BUBBU)	76	91	TKIPAVFKIDALNENK	0,00E+00	0,00E+00	0,00E+00	8,25E+06	0,00E+00	5,77E+06	0,00E+00	1,47E+08	1,62E+07
Beta-lactoglobulin (P02754 · LACB_BOVIN; A0A7M1I6E3_BUBBU)	78	91	IPAVFKIDALNENK	3,66E+07	1,36E+07	4,15E+07	2,62E+07	1,91E+07	1,97E+07	4,88E+07	2,76E+08	3,27E+07
Beta-lactoglobulin (P02754 · LACB_BOVIN; A0A7M1I6E3_BUBBU)	78	101	IPAVFKIDALNENKVLVLDTDYKK	0,00E+00	0,00E+00	0,00E+00	0,00E+00	0,00E+00	0,00E+00	0,00E+00	2,71E+06	0,00E+00

Beta-lactoglobulin (P02754 · LACB_BOVIN; A0A7M1I6E3_BUBBU)	84	91	IDALNENK	4,21E+10	4,66E+10	3,53E+10	3,73E+10	4,43E+10	4,11E+10	6,97E+10	4,28E+10	2,47E+10
Beta-lactoglobulin (P02754 · LACB_BOVIN; A0A7M1I6E3_BUBBU)	84	100	IDALNENKVLVLDTDYK	1,04E+08	1,17E+08	1,01E+08	4,55E+07	5,79E+07	4,93E+07	9,89E+07	6,53E+07	1,08E+08
Beta-lactoglobulin (P02754 · LACB_BOVIN; A0A7M1I6E3_BUBBU)	84	101	IDALNENKVLVLDTDYKK	0,00E+00	0,00E+00	0,00E+00	0,00E+00	0,00E+00	0,00E+00	0,00E+00	2,09E+08	3,49E+07
Beta-lactoglobulin (P02754 · LACB_BOVIN; A0A7M1I6E3_BUBBU)	92	100	VLVLDTDYK	1,08E+11	1,19E+11	1,17E+11	1,03E+11	1,11E+11	1,03E+11	1,13E+11	8,07E+10	1,75E+11
Beta-lactoglobulin (P02754 · LACB_BOVIN; A0A7M1I6E3_BUBBU)	92	101	VLVLDTDYKK	0,00E+00	0,00E+00	0,00E+00	1,97E+09	2,35E+09	6,94E+08	0,00E+00	1,22E+09	6,21E+08
Beta-lactoglobulin (P02754 · LACB_BOVIN; A0A7M1I6E3_BUBBU)	101	124	KYLLFCMENSAEPEQSLACQCLVR	0,00E+00	0,00E+00	0,00E+00	0,00E+00	0,00E+00	0,00E+00	0,00E+00	2,61E+06	0,00E+00
Beta-lactoglobulin (P02754 · LACB_BOVIN; A0A7M1I6E3_BUBBU)	102	124	YLLFCMENSAEPEQSLACQCLVR	0,00E+00	0,00E+00	0,00E+00	5,05E+06	0,00E+00	0,00E+00	0,00E+00	2,32E+07	0,00E+00
Beta-lactoglobulin (P02754 · LACB_BOVIN; A0A7M1I6E3_BUBBU)	125	135	TPEVDDEALEK	2,24E+10	2,40E+10	1,71E+10	2,07E+10	1,62E+10	2,46E+10	4,47E+10	1,70E+10	8,66E+09
Beta-lactoglobulin (P02754 · LACB_BOVIN; A0A7M1I6E3_BUBBU)	125	138	TPEVDDEALEKFDK	0,00E+00	0,00E+00	2,55E+06	0,00E+00	0,00E+00	4,08E+06	0,00E+00	1,47E+06	3,95E+09
Beta-lactoglobulin (P02754 · LACB_BOVIN; A0A7M1I6E3_BUBBU)	125	141	TPEVDDEALEKFDKALK	3,64E+06	1,81E+06	1,86E+06	1,61E+06	1,16E+06	6,77E+06	0,00E+00	3,25E+06	0,00E+00
Beta-lactoglobulin (P02754 · LACB_BOVIN; A0A7M1I6E3_BUBBU)	142	148	ALPMHIR	1,19E+10	1,04E+10	1,38E+10	1,09E+10	1,13E+10	1,07E+10	5,21E+09	9,52E+09	2,02E+10
Butyrophilin subfamily 1 member A1 (P18892 · BT1A1_BOVIN; M9WP41_BUBBU)	70	78	VSPAVFVSR	1,45E+07	1,49E+07	0,00E+00	0,00E+00	0,00E+00	0,00E+00	2,47E+07	0,00E+00	8,84E+07
Butyrophilin subfamily 1 member A1 (P18892 · BT1A1_BOVIN; M9WP41_BUBBU)	95	110	VSLVEDHIAEGSVAVR	0,00E+00	0,00E+00	0,00E+00	0,00E+00	0,00E+00	0,00E+00	6,11E+06	0,00E+00	0,00E+00
Butyrophilin subfamily 1 member A1 (P18892 ·	182	192	GEEFPSMSESR	0,00E+00	0,00E+00	0,00E+00	0,00E+00	0,00E+00	0,00E+00	0,00E+00	1,34E+06	0,00E+00

BT1A1_BOVIN; M9WP41_BUBBU)												
Butyrophilin subfamily 1 member A1 (P18892 · BT1A1_BOVIN; M9WP41_BUBBU)	193	203	NPDEEGLFTVR	1,02E+07	1,01E+07	8,31E+06	2,79E+06	0,00E+00	5,61E+06	1,57E+07	6,92E+06	1,45E+07
Butyrophilin subfamily 1 member A1 (P18892 · BT1A1_BOVIN; M9WP41_BUBBU)	222	229	NLLLGQEK	7,03E+06	0,00E+00	0,00E+00	1,81E+07	1,19E+07	1,73E+07	4,90E+06	2,18E+07	1,99E+07
Butyrophilin subfamily 1 member A1 (P18892 · BT1A1_BOVIN; M9WP41_BUBBU)	341	350	FDSWPCVMGR	0,00E+00	0,00E+00	0,00E+00	0,00E+00	1,28E+06	0,00E+00	0,00E+00	1,46E+06	0,00E+00
Butyrophilin subfamily 1 member A1 (P18892 · BT1A1_BOVIN; M9WP41_BUBBU)	358	367	HYWEVEVGDR	0,00E+00	0,00E+00	0,00E+00	3,07E+06	5,08E+06	3,04E+06	0,00E+00	4,09E+06	0,00E+00
Butyrophilin subfamily 1 member A1 (P18892 · BT1A1_BOVIN; M9WP41_BUBBU)	411	420	TPLPLAGPPR	8,56E+06	8,08E+06	6,56E+06	0,00E+00	0,00E+00	0,00E+00	0,00E+00	6,40E+06	0,00E+00
Butyrophilin subfamily 1 member A1 (P18892 · BT1A1_BOVIN; M9WP41_BUBBU)	494	515	EIPLSPMGEDSASGDIETLHSK	0,00E+00	0,00E+00	0,00E+00	0,00E+00	0,00E+00	0,00E+00	0,00E+00	0,00E+00	5,65E+06
Cathepsin D (P80209 · CATD_BOVIN)	35	43	YATGEPAVR	3,89E+05	4,72E+05	0,00E+00	0,00E+00	0,00E+00	0,00E+00	0,00E+00	0,00E+00	0,00E+00
Cathepsin D (P80209 · CATD_BOVIN)	371	377	YYTVFDR	4,21E+06	2,34E+06	8,39E+05	0,00E+00	0,00E+00	0,00E+00	0,00E+00	0,00E+00	0,00E+00
Fatty acid-binding protein, heart (Q5XLB1_BUBBU)	2	10	VDAFVGTWK	0,00E+00	0,00E+00	0,00E+00	0,00E+00	0,00E+00	0,00E+00	0,00E+00	1,50E+07	0,00E+00
Fatty acid-binding protein, heart (Q5XLB1_BUBBU)	16	22	NFDDYMK	7,14E+07	4,25E+07	2,73E+07	3,24E+07	5,00E+07	5,03E+07	0,00E+00	3,57E+07	0,00E+00
Fatty acid-binding protein, heart (Q5XLB1_BUBBU)	23	31	SLGVGFATR	1,64E+09	1,62E+09	1,74E+09	1,30E+09	1,12E+09	1,03E+09	1,24E+07	1,39E+09	1,96E+07
Fatty acid-binding protein, heart (Q5XLB1_BUBBU)	60	66	NTEISFK	6,57E+08	7,50E+08	6,04E+08	6,36E+08	2,06E+08	3,48E+08	0,00E+00	2,25E+08	0,00E+00
Fatty acid-binding protein, heart (Q5XLB1_BUBBU)	67	79	LGVEFDETTADDR	1,88E+08	2,14E+08	1,48E+08	1,39E+08	1,21E+08	1,24E+08	0,00E+00	1,42E+08	0,00E+00
Fatty acid-binding protein, heart (Q5XLB1_BUBBU)	83	91	SIVTLDGGK	8,05E+08	9,45E+08	7,77E+08	7,77E+08	7,70E+08	5,89E+08	0,00E+00	7,92E+08	0,00E+00
Fatty acid-binding protein, heart (Q5XLB1_BUBBU)	98	107	WNGQETSLVR	7,87E+08	8,11E+08	8,17E+08	6,79E+08	6,10E+08	5,77E+08	0,00E+00	6,19E+08	0,00E+00

Glycosylation-dependent cell adhesion molecule 1 (P80195 · GLCM1_BOVIN)	125	134	IMRNLENTVK	9,13E+06	1,73E+07	0,00E+00	0,00E+00	5,00E+06	5,55E+06	0,00E+00	1,67E+07	0,00E+00
Glycosylation-dependent cell adhesion molecule 1 (P80195 · GLCM1_BOVIN)	128	134	NLENTVK	0,00E+00	0,00E+00	0,00E+00	0,00E+00	0,00E+00	0,00E+00	7,60E+06	0,00E+00	0,00E+00
Glycosylation-dependent cell adhesion molecule 1 (A0A6F8V7K3_BUBBU)	95	117	NAALGSEETTEHTPSDASTTEGK	5,51E+08	5,81E+08	4,07E+08	1,07E+09	9,20E+08	8,47E+08	1,81E+07	1,05E+09	1,78E+09
Glycosylation-dependent cell adhesion molecule 1 (A0A6F8V7K3_BUBBU)	95	124	NAALGSEETTEHTPSDASTTEGKLMELGHK	0,00E+00	0,00E+00	0,00E+00	0,00E+00	0,00E+00	0,00E+00	0,00E+00	0,00E+00	1,86E+07
Glycosylation-dependent cell adhesion molecule 1 (A0A6F8V7K3_BUBBU)	125	134	IMTNLENTVK	1,40E+09	1,42E+09	1,25E+09	1,68E+09	1,26E+09	9,63E+08	2,77E+07	1,88E+09	3,20E+10
Glycosylation-dependent cell adhesion molecule 1 (A0A6F8V7K3_BUBBU)	125	138	IMTNLENTVKETIK	0,00E+00	0,00E+00	0,00E+00	0,00E+00	0,00E+00	0,00E+00	0,00E+00	0,00E+00	4,12E+08
Glycosylation-dependent cell adhesion molecule 1 (A0A6F8V7K3_BUBBU)	125	141	IMTNLENTVKETIKYLK	0,00E+00	0,00E+00	0,00E+00	0,00E+00	0,00E+00	0,00E+00	0,00E+00	0,00E+00	2,65E+07
Glycosylation-dependent cell adhesion molecule 1 (A0A6F8V7K3_BUBBU)	142	153	SLFSHASEVVKP	6,55E+08	5,87E+08	6,61E+08	2,64E+09	1,92E+09	1,57E+09	2,10E+07	2,47E+09	2,30E+10
Glycosylation-dependent cell adhesion molecule 1 (P80195 · GLCM1_BOVIN; A0A6F8V7K3_BUBBU)	43	53	NLQISNEDLSK	4,53E+09	4,79E+09	4,34E+09	1,85E+09	1,68E+09	1,30E+09	1,33E+09	1,27E+09	3,48E+09
Glycosylation-dependent cell adhesion molecule 1 (P80195 · GLCM1_BOVIN; A0A6F8V7K3_BUBBU)	118	124	LMELGHK	4,45E+08	3,72E+08	2,22E+08	2,42E+08	3,09E+08	4,04E+08	9,94E+05	3,79E+08	5,69E+09
Immunoglobulin light chain, lambda gene cluster (Q1RMN8_BOVIN)	37	50	VSITCSGSSSNIGR	1,64E+08	1,44E+08	1,82E+08	1,26E+08	1,31E+08	1,22E+08	3,89E+07	7,96E+07	0,00E+00
Immunoglobulin light chain, lambda gene cluster (Q1RMN8_BOVIN)	133	151	SPPSVTLFPPSTEELNGNK	0,00E+00	0,00E+00	0,00E+00	0,00E+00	7,68E+06	1,95E+07	1,46E+08	0,00E+00	0,00E+00
Immunoglobulin light chain, lambda gene cluster (Q1RMN8_BOVIN)	172	179	ADGSTITR	1,20E+07	2,33E+07	1,65E+07	2,97E+07	3,31E+07	3,78E+07	3,82E+07	2,94E+07	0,00E+00
Immunoglobulin light chain, lambda gene cluster (Q1RMN8_BOVIN)	194	208	YAASSYLSLTSSDWK	1,21E+06	9,17E+06	8,14E+06	2,36E+07	5,42E+07	1,04E+08	2,06E+08	0,00E+00	0,00E+00

Immunoglobulin light chain, lambda gene cluster (Q1RMN8_BOVIN)	209	226	SKGSYSCEVTHEGSTVTK	0,00E+00	0,00E+00	1,47E+06	3,93E+07	4,61E+07	3,89E+07	0,00E+00	4,35E+07	0,00E+00
Immunoglobulin light chain, lambda gene cluster (Q1RMN8_BOVIN)	211	226	GSYSCEVTHEGSTVTK	4,11E+09	4,11E+09	3,60E+09	2,76E+09	3,05E+09	2,67E+09	1,68E+09	2,37E+09	0,00E+00
Immunoglobulin light chain, lambda gene cluster (Q1RMN8_BOVIN)	227	234	TVKPSECS	0,00E+00	0,00E+00	1,50E+07	0,00E+00	0,00E+00	0,00E+00	0,00E+00	0,00E+00	0,00E+00
Immunoglobulin light chain, lambda gene cluster (Q1RMN8_BOVIN)	252	266	AEVLSPVVSVFVPPR	0,00E+00	0,00E+00	0,00E+00	0,00E+00	0,00E+00	0,00E+00	3,84E+06	0,00E+00	0,00E+00
Kappa-casein (P02668 · CASK_BOVIN)	1	10	QEQNQEQPIR	2,55E+07	1,50E+07	1,78E+07	2,13E+07	2,67E+07	2,86E+07	4,12E+07	2,90E+07	3,30E+06
Kappa-casein (P02668 · CASK_BOVIN)	69	86	SPAQILQWQVLSNTVPAK	0,00E+00	0,00E+00	1,38E+06	2,41E+06	3,59E+06	7,35E+06	8,50E+07	0,00E+00	0,00E+00
Kappa-casein (P02668 · CASK_BOVIN)	87	97	SCQAQPTTMAR	0,00E+00	2,70E+06	3,56E+06	1,15E+07	2,42E+07	5,93E+07	2,73E+08	0,00E+00	0,00E+00
Kappa-casein (Q712N6_BUBBU)	25	34	YIPIQYVLSR	0,00E+00	0,00E+00	0,00E+00	0,00E+00	0,00E+00	0,00E+00	0,00E+00	0,00E+00	8,42E+07
Kappa-casein (Q712N6_BUBBU)	38	45	FFNDKIAK	0,00E+00	0,00E+00	0,00E+00	0,00E+00	0,00E+00	0,00E+00	0,00E+00	0,00E+00	6,88E+07
Kappa-casein (Q712N6_BUBBU)	90	107	SPAQILQWQVLPNTVPAK	0,00E+00	0,00E+00	0,00E+00	1,45E+06	0,00E+00	0,00E+00	0,00E+00	1,53E+06	9,14E+07
Kappa-casein (Q712N6_BUBBU)	98	111	HPHPHLSFMAIPPK	0,00E+00	0,00E+00	0,00E+00	0,00E+00	0,00E+00	3,31E+06	0,00E+00	0,00E+00	0,00E+00
Kappa-casein (Q712N6_BUBBU)	108	118	SCQAQPTTMTR	4,92E+07	8,11E+07	6,17E+07	1,58E+08	1,84E+08	1,46E+08	0,00E+00	1,76E+08	0,00E+00
Lactoferrin (B1PZZ8_BUBBU)	1	8	ALFGENGK	0,00E+00	6,77E+06	0,00E+00	0,00E+00	7,25E+06	0,00E+00	0,00E+00	0,00E+00	0,00E+00
Lactoferrin (Q0PGA3_BUBBU)	1	8	FGSPPGQR	7,56E+06	9,97E+06	3,04E+06	0,00E+00	5,44E+06	1,75E+06	8,85E+06	4,56E+06	0,00E+00
Lactoferrin (Q0PGA4_BUBBU)	18	26	LRPVAAEIY	0,00E+00	0,00E+00	0,00E+00	0,00E+00	0,00E+00	0,00E+00	0,00E+00	0,00E+00	4,22E+07
Lactoperoxidase (P80025 · PERL_BOVIN)	58	75	TTLSSEAPTTQQLSEYFK	5,05E+05	0,00E+00	0,00E+00	1,14E+06	0,00E+00	0,00E+00	0,00E+00	7,69E+06	0,00E+00
Lactoperoxidase (P80025 · PERL_BOVIN)	87	96	NGQVWEESLK	0,00E+00	0,00E+00	0,00E+00	0,00E+00	0,00E+00	0,00E+00	4,25E+06	0,00E+00	0,00E+00
Lactoperoxidase (P80025 · PERL_BOVIN)	140	148	TITGDCNNR	0,00E+00	0,00E+00	0,00E+00	4,66E+06	4,02E+06	5,07E+06	2,12E+06	1,22E+07	0,00E+00
Lactoperoxidase (P80025 · PERL_BOVIN)	150	158	SPALGAANR	0,00E+00	0,00E+00	3,25E+07	5,50E+07	4,41E+07	2,25E+07	0,00E+00	0,00E+00	0,00E+00
Lactoperoxidase (P80025 · PERL_BOVIN)	163	181	WLPAEYEDGLALPFGWTQR	0,00E+00	0,00E+00	0,00E+00	0,00E+00	0,00E+00	0,00E+00	0,00E+00	9,24E+05	0,00E+00

Lactoperoxidase (P80025 · PERL_BOVIN)	199	213	IVGYLDEEGVLDQNR	0,00E+00	0,00E+00	0,00E+00	0,00E+00	0,00E+00	0,00E+00	7,30E+06	0,00E+00	0,00E+00
Lactoperoxidase (P80025 · PERL_BOVIN)	244	263	TQCEEYCIQGDNCFPIMFPK	0,00E+00	0,00E+00	0,00E+00	0,00E+00	0,00E+00	0,00E+00	0,00E+00	1,34E+06	0,00E+00
Lactoperoxidase (P80025 · PERL_BOVIN)	280	294	AGFVCPTPPYQSLAR	1,74E+08	1,78E+08	2,07E+08	9,84E+07	7,89E+07	7,61E+07	3,66E+07	1,10E+08	0,00E+00
Lactoperoxidase (P80025 · PERL_BOVIN)	295	319	EQINAVTSFLDASLVYGSEPSLASR	0,00E+00	0,00E+00	0,00E+00	0,00E+00	0,00E+00	0,00E+00	0,00E+00	5,16E+05	0,00E+00
Lactoperoxidase (P80025 · PERL_BOVIN)	363	372	VPCFLAGDFR	2,02E+07	2,19E+07	1,30E+07	1,47E+07	2,58E+07	2,63E+07	7,10E+06	1,35E+07	0,00E+00
Lactoperoxidase (P80025 · PERL_BOVIN)	373	388	ASEQILLATAHTLLLR	0,00E+00	0,00E+00	0,00E+00	0,00E+00	0,00E+00	0,00E+00	0,00E+00	9,26E+05	0,00E+00
Lactoperoxidase (P80025 · PERL_BOVIN)	400	408	LNPHWNGEK	0,00E+00	0,00E+00	0,00E+00	0,00E+00	0,00E+00	0,00E+00	1,09E+06	0,00E+00	0,00E+00
Lactoperoxidase (P80025 · PERL_BOVIN)	428	440	DYLPIVLGSEMQK	1,35E+07	4,63E+07	1,50E+07	2,33E+07	1,24E+07	0,00E+00	0,00E+00	0,00E+00	0,00E+00
Lactoperoxidase (P80025 · PERL_BOVIN)	456	465	ISNVFTFAFR	0,00E+00	0,00E+00	0,00E+00	0,00E+00	0,00E+00	0,00E+00	0,00E+00	2,40E+06	0,00E+00
Lactoperoxidase (P80025 · PERL_BOVIN)	466	477	FGHMEVPSTVSR	3,56E+08	3,51E+08	2,94E+08	2,22E+08	2,19E+08	2,06E+08	5,75E+07	1,55E+08	0,00E+00
Lactoperoxidase (P80025 · PERL_BOVIN)	506	514	DGGIDPLVR	1,44E+08	1,59E+08	1,53E+08	9,54E+07	9,90E+07	8,56E+07	6,66E+07	1,45E+08	0,00E+00
Lactoperoxidase (P80025 · PERL_BOVIN)	529	535	MVTSELR	1,83E+08	1,83E+08	1,42E+08	2,26E+06	0,00E+00	0,00E+00	0,00E+00	2,20E+06	0,00E+00
Lactoperoxidase (P80025 · PERL_BOVIN)	538	544	LFQPTHK	7,70E+07	8,02E+07	4,44E+07	0,00E+00	0,00E+00	0,00E+00	0,00E+00	6,79E+07	0,00E+00
Lactoperoxidase (P80025 · PERL_BOVIN)	545	557	IHGFDLAAINLQR	2,41E+08	2,62E+08	2,64E+08	2,07E+08	1,69E+08	1,67E+08	6,46E+07	2,52E+08	0,00E+00
Lactoperoxidase (P80025 · PERL_BOVIN)	560	570	DHGMPGYNSWR	4,84E+07	4,13E+07	4,58E+07	2,65E+07	2,56E+07	2,97E+07	4,37E+06	3,47E+07	0,00E+00
Lactoperoxidase (P80025 · PERL_BOVIN)	571	579	GFCGLSQPK	2,40E+08	1,96E+08	3,93E+08	5,57E+08	0,00E+00	2,96E+08	0,00E+00	5,04E+08	0,00E+00
Lactoperoxidase (P80025 · PERL_BOVIN)	603	621	TPDNIDIWIGGNAEPMVER	0,00E+00	0,00E+00	0,00E+00	0,00E+00	0,00E+00	0,00E+00	0,00E+00	8,63E+05	0,00E+00
Lactoperoxidase (P80025 · PERL_BOVIN)	624	634	VGPLLACLLGR	0,00E+00	0,00E+00	5,78E+06	0,00E+00	0,00E+00	0,00E+00	0,00E+00	1,42E+06	0,00E+00
Lactoperoxidase (P80025 · PERL_BOVIN)	645	656	FWWENPGVFTEK	3,84E+08	4,23E+08	3,98E+08	3,05E+08	2,33E+08	2,45E+08	8,54E+07	2,64E+08	0,00E+00
Lactoperoxidase (P80025 · PERL_BOVIN)	669	678	LICDNTHITK	1,84E+09	1,80E+09	1,45E+09	1,44E+09	1,42E+09	1,15E+09	3,84E+08	1,44E+09	0,00E+00
Lactoperoxidase (P80025 · PERL_BOVIN)	702	710	LDLSPWASR	1,34E+08	4,21E+08	1,67E+08	2,34E+08	9,96E+07	1,09E+08	0,00E+00	3,43E+08	2,23E+07
Lactotransferrin (P24627 · TRFL_BOVIN)	59	66	AFALECIR	0,00E+00	7,07E+06	1,37E+07	1,55E+07	0,00E+00	2,26E+07	2,99E+08	0,00E+00	0,00E+00

Lactotransferrin (P24627 · TRFL_BOVIN)	171	182	FFSASCVPCIDR	0,00E+00	2,78E+06	6,03E+06	8,66E+06	1,03E+07	2,66E+07	1,51E+08	0,00E+00	0,00E+00
Lactotransferrin (P24627 · TRFL_BOVIN)	406	423	GEADALNLDGGYIYTAGK	0,00E+00	0,00E+00	0,00E+00	0,00E+00	0,00E+00	1,13E+07	8,00E+07	0,00E+00	0,00E+00
Lactotransferrin (P24627 · TRFL_BOVIN)	598	619	KPVTEAQSCHLAVAPNHAVVSR	0,00E+00	0,00E+00	0,00E+00	3,00E+06	1,48E+07	7,67E+06	5,48E+07	0,00E+00	0,00E+00
Lactotransferrin (P24627 · TRFL_BOVIN)	628	639	QVLLHQQALFGK	0,00E+00	0,00E+00	0,00E+00	0,00E+00	0,00E+00	8,91E+06	1,04E+08	0,00E+00	0,00E+00
Lactotransferrin (P24627 · TRFL_BOVIN)	27	37	WCTISQPEWFK	0,00E+00	0,00E+00	0,00E+00	0,00E+00	1,30E+07	1,72E+07	1,09E+08	0,00E+00	0,00E+00
Lactotransferrin (A0A387L9I6_BUBBU)	171	182	FFSASCVPCVDR	2,23E+08	2,38E+08	2,65E+08	2,11E+08	1,79E+08	1,70E+08	0,00E+00	2,64E+08	0,00E+00
Lactotransferrin (A0A387L9I6_BUBBU)	352	360	ETAEEVQAR	7,59E+06	8,11E+06	0,00E+00	2,07E+06	1,95E+06	4,74E+06	0,00E+00	2,81E+06	0,00E+00
Lactotransferrin (A0A387L9I6_BUBBU)	406	423	GEADALSLDGGYIYTAGK	2,02E+07	2,98E+07	2,49E+07	3,74E+07	3,34E+07	2,94E+07	0,00E+00	3,63E+07	2,92E+07
Lactotransferrin (A0A387L9I6_BUBBU)	302	315	SGSFQLFGSPPGQR	1,92E+08	2,09E+08	2,06E+08	1,56E+08	1,28E+08	1,14E+08	0,00E+00	1,56E+08	0,00E+00
Lactotransferrin (A0A387L9I6_BUBBU)	59	66	AFVLECIR	1,58E+08	2,57E+08	2,97E+08	2,46E+08	1,98E+08	1,87E+08	0,00E+00	2,90E+08	0,00E+00
Lactotransferrin (A0A387L9I6_BUBBU)	27	37	WCTISQPEWLK	5,78E+07	6,95E+07	1,91E+07	6,38E+07	7,77E+07	6,90E+07	0,00E+00	5,63E+07	0,00E+00
Lactotransferrin (A0A387L9I6_BUBBU)	194	205	GEGENQCACSPR	4,76E+06	4,80E+06	2,58E+06	5,17E+06	6,06E+06	5,11E+06	0,00E+00	7,25E+06	0,00E+00
Lactotransferrin (A0A387L9I6_BUBBU)	623	642	AAHVEQVLLHQQALFGENGK	2,36E+08	2,15E+08	2,41E+08	1,76E+08	1,51E+08	1,34E+08	0,00E+00	2,62E+08	9,68E+07
Lactotransferrin (A0A387L9I6_BUBBU)	598	622	KPVTEAQSCHLAVAPNHAVVSLSER	1,91E+07	1,30E+07	5,08E+07	2,55E+07	2,69E+07	2,46E+07	0,00E+00	2,48E+07	0,00E+00
Lactotransferrin (P24627 · TRFL_BOVIN; G9DAR3_BUBBU)	206	216	EPYFGYSGAFK	1,03E+07	2,48E+07	2,04E+07	2,66E+07	3,58E+07	4,35E+07	5,68E+06	5,83E+06	7,20E+06
Lactotransferrin (P24627 · TRFL_BOVIN; G9DAR3_BUBBU)	439	459	HSSLDCVLRPTEGYLAVAVVK	1,82E+07	3,04E+07	2,93E+07	3,37E+07	2,21E+07	2,85E+07	3,42E+07	3,61E+07	0,00E+00
Lactotransferrin (P24627 · TRFL_BOVIN; G9DAR3_BUBBU)	364	375	VVWCAVGPEEQK	1,02E+08	1,19E+08	1,26E+08	1,31E+08	1,20E+08	1,18E+08	9,79E+07	1,31E+08	0,00E+00
Lactotransferrin (P24627 · TRFL_BOVIN; G9DAR3_BUBBU)	424	434	CGLVPVLAENR	2,77E+06	5,56E+06	0,00E+00	2,61E+06	0,00E+00	2,49E+06	0,00E+00	3,24E+06	0,00E+00
Lactotransferrin (P24627 · TRFL_BOVIN; G9DAR3_BUBBU)	230	240	ETTVFENLPEK	1,68E+08	1,09E+08	1,45E+08	1,67E+08	1,51E+08	1,71E+08	1,75E+08	1,58E+08	6,41E+07

Lactotransferrin (P24627 · TRFL_BOVIN; G9DAR3_BUBBU)	93	104	LRPVAAEIYGTK	1,54E+09	1,47E+09	1,57E+09	1,63E+09	1,36E+09	1,34E+09	1,05E+09	1,22E+09	7,68E+08
Lactotransferrin (P24627 · TRFL_BOVIN; G9DAR3_BUBBU)	183	193	QAYPNLCQLCK	0,00E+00	0,00E+00	4,73E+06	2,23E+06	0,00E+00	0,00E+00	0,00E+00	3,63E+06	0,00E+00
Lactotransferrin (P24627 · TRFL_BOVIN; G9DAR3_BUBBU)	590	597	LLCLDGTR	6,54E+08	6,34E+08	4,23E+08	6,61E+08	5,58E+08	4,54E+08	6,29E+08	4,47E+08	0,00E+00
Lactotransferrin (P24627 · TRFL_BOVIN; G9DAR3_BUBBU)	670	692	LGGRPTYEEYLGTEYVTAIANLK	0,00E+00	0,00E+00	0,00E+00	0,00E+00	0,00E+00	0,00E+00	0,00E+00	1,67E+06	0,00E+00
Lactotransferrin (P24627 · TRFL_BOVIN; G9DAR3_BUBBU)	657	669	NLLFNDNTECLAK	2,31E+08	2,35E+08	2,26E+08	2,18E+08	2,30E+08	2,00E+08	2,28E+08	2,13E+08	0,00E+00
Lactotransferrin (P24627 · TRFL_BOVIN; G9DAR3_BUBBU)	105	118	ESPQTHYYAVAVVK	3,30E+08	3,10E+08	3,71E+08	4,24E+08	3,91E+08	3,86E+08	2,02E+08	2,98E+08	1,11E+08
Lactotransferrin (P24627 · TRFL_BOVIN; G9DAR3_BUBBU)	263	277	ECHLAQVPSHAVVAR	7,35E+08	7,08E+08	7,47E+08	9,00E+08	7,38E+08	7,50E+08	4,77E+08	7,77E+08	0,00E+00
Lactotransferrin (P24627 · TRFL_BOVIN; G9DAR3_BUBBU)	120	131	GSNFQLDQLQGR	3,01E+08	1,71E+08	2,78E+08	3,28E+08	3,11E+08	1,36E+08	3,87E+08	4,20E+08	2,34E+08
Lactotransferrin (P24627 · TRFL_BOVIN; G9DAR3_BUBBU)	119	131	KGSNFQLDQLQGR	0,00E+00	6,90E+06	4,14E+07	7,25E+06	0,00E+00	0,00E+00	9,28E+06	0,00E+00	1,53E+08
Lactotransferrin (P24627 · TRFL_BOVIN; G9DAR3_BUBBU)	304	315	SFQLFGSPPGQR	0,00E+00	0,00E+00	0,00E+00	0,00E+00	0,00E+00	0,00E+00	3,47E+05	0,00E+00	0,00E+00
Lactotransferrin (P24627 · TRFL_BOVIN; G9DAR3_BUBBU)	329	342	IPSKVDSALYLGSR	0,00E+00	0,00E+00	0,00E+00	0,00E+00	0,00E+00	0,00E+00	0,00E+00	0,00E+00	4,58E+06
Lactotransferrin (P24627 · TRFL_BOVIN; G9DAR3_BUBBU)	321	328	DSALGFLR	1,55E+08	1,56E+08	1,54E+08	1,42E+08	1,24E+08	1,44E+08	1,09E+08	1,28E+08	9,39E+07
Lactotransferrin (P24627 · TRFL_BOVIN; G9DAR3_BUBBU)	333	342	VDSALYLGSR	7,56E+08	7,51E+08	7,83E+08	0,00E+00	0,00E+00	1,97E+08	1,97E+08	4,28E+08	1,58E+08
Lactotransferrin (P24627 · TRFL_BOVIN; G9DAR3_BUBBU)	217	229	CLQDGAGDVAFVK	3,48E+07	3,78E+07	3,44E+07	3,59E+07	3,66E+07	3,63E+07	3,53E+07	4,10E+07	0,00E+00

Lactotransferrin (P24627 · TRFL_BOVIN; G9DAR3_BUBBU)	520	533	LCALCAGDDQGLDK	1,99E+07	2,30E+07	3,48E+07	3,27E+07	2,70E+07	2,80E+07	1,78E+07	3,25E+07	0,00E+00
Lactotransferrin (P24627 · TRFL_BOVIN; G9DAR3_BUBBU)	551	563	CLAEDVGDVAFVK	0,00E+00	2,46E+06	5,46E+06	2,48E+06	5,66E+06	5,07E+06	2,32E+06	2,66E+06	0,00E+00
Lactotransferrin (P24627 · TRFL_BOVIN; G9DAR3_BUBBU)	48	57	LGAPSITCVR	6,66E+08	7,34E+08	6,83E+08	4,86E+08	4,18E+08	3,81E+08	5,36E+08	4,43E+08	0,00E+00
L-lactate dehydrogenase (K0IT60_BUBBU)	1	7	MATLKEK	0,00E+00	0,00E+00	0,00E+00	0,00E+00	0,00E+00	7,57E+07	0,00E+00	0,00E+00	0,00E+00
L-lactate dehydrogenase (K0IT60_BUBBU)	159	170	VIGSGCNLDSAR	0,00E+00	0,00E+00	0,00E+00	0,00E+00	0,00E+00	0,00E+00	0,00E+00	0,00E+00	0,00E+00
Milk fat globule EGF factor 8 protein (A0A5J6SHA6_BUBBU)	225	236	IFVGNVNNSGLK	3,21E+06	0,00E+00	2,96E+06	0,00E+00	0,00E+00	0,00E+00	0,00E+00	0,00E+00	0,00E+00
Milk fat globule EGF factor 8 protein (A0A5J6SHA6_BUBBU; MFGM_BOVIN)	343	352	VTGIITQGAR	1,47E+07	0,00E+00	7,26E+06	0,00E+00	0,00E+00	0,00E+00	0,00E+00	0,00E+00	0,00E+00
Milk fat globule EGF factor 8 protein (A0A5J6SHA6_BUBBU; MFGM_BOVIN)	400	409	NIFETPFQAR	7,94E+05	0,00E+00	0,00E+00	0,00E+00	0,00E+00	0,00E+00	0,00E+00	0,00E+00	0,00E+00
Osteopontin (P31096 · OSTP_BOVIN)	1	14	LPVKPTSSGSSEEK	8,89E+06	8,03E+06	4,88E+06	6,91E+06	9,32E+06	8,13E+06	0,00E+00	7,69E+06	8,06E+06
Osteopontin (P31096 · OSTP_BOVIN; Q1ZZS7_BUBBU)	20	35	YPDAVATWLKPDPSQK	0,00E+00	0,00E+00	0,00E+00	0,00E+00	0,00E+00	0,00E+00	0,00E+00	0,00E+00	5,34E+07
Osteopontin (P31096 · OSTP_BOVIN; Q1ZZS7_BUBBU)	137	145	GDSVAYGLK	1,07E+07	7,99E+06	4,87E+06	0,00E+00	0,00E+00	0,00E+00	3,30E+06	0,00E+00	0,00E+00
Osteopontin (P31096 · OSTP_BOVIN; Q1ZZS7_BUBBU)	182	190	TSQLTDHSK	0,00E+00	1,12E+06	1,04E+06	4,90E+05	8,78E+05	0,00E+00	0,00E+00	0,00E+00	0,00E+00
Osteopontin (P31096 · OSTP_BOVIN; Q1ZZS7_BUBBU)	211	222	HSNLIESQENSK	3,06E+08	3,74E+08	2,41E+08	2,25E+08	2,77E+08	2,72E+08	4,31E+08	2,29E+08	2,06E+08
Osteopontin (P31096 · OSTP_BOVIN; Q1ZZS7_BUBBU)	223	233	LSQEFHSLEDK	8,53E+08	8,57E+08	8,76E+08	5,54E+08	5,90E+08	5,60E+08	7,94E+08	5,44E+08	9,00E+08
Osteopontin (P31096 · OSTP_BOVIN; Q1ZZS7_BUBBU)	223	239	LSQEFHSLEDKLDLDHK	1,97E+06	0,00E+00	0,00E+00	0,00E+00	0,00E+00	0,00E+00	0,00E+00	0,00E+00	2,36E+07
Osteopontin (P31096 · OSTP_BOVIN; Q1ZZS7_BUBBU)	250	262	ISHELDSASSEVN	3,91E+07	5,37E+07	4,00E+07	4,52E+07	5,65E+07	6,38E+07	1,97E+08	3,11E+07	1,91E+07

Perilipin-2 (Q9TUM6 · PLIN2_BOVIN)	94	109	LPILNQPTNQVVANAK	0,00E+00	0,00E+00	0,00E+00	7,31E+05	0,00E+00	0,00E+00	0,00E+00	7,85E+05	0,00E+00
Perilipin-2 (Q9TUM6 · PLIN2_BOVIN)	183	195	SELLVDQYLPLTK	0,00E+00	0,00E+00	4,31E+05	0,00E+00	0,00E+00	0,00E+00	0,00E+00	0,00E+00	0,00E+00
Perilipin-2 (Q9TUM6 · PLIN2_BOVIN; A0A097P7B0_BUBBU)	117	127	DAVTTTVTGAK	4,61E+07	5,26E+07	4,58E+07	4,66E+07	4,64E+07	4,74E+07	3,12E+07	5,10E+07	7,03E+07
Perilipin-2 (Q9TUM6 · PLIN2_BOVIN; A0A097P7B0_BUBBU)	128	140	DSVASTITGVVDR	8,48E+06	8,96E+06	4,01E+06	0,00E+00	0,00E+00	0,00E+00	2,54E+06	0,00E+00	7,71E+07
Plasminogen (P06868 · PLMN_BOVIN)	289	305	NYGGTVAVTESGHTCQR	0,00E+00	0,00E+00	0,00E+00	0,00E+00	0,00E+00	0,00E+00	2,82E+06	0,00E+00	0,00E+00
Plasminogen (P06868 · PLMN_BOVIN)	394	403	GTSSTTITGR	0,00E+00	0,00E+00	0,00E+00	0,00E+00	0,00E+00	0,00E+00	3,04E+05	0,00E+00	0,00E+00
Polymeric immunoglobulin receptor (P81265 · PIGR_BOVIN)	20	34	SPIFGPEEVTSVEGR	1,64E+08	1,70E+08	1,52E+08	1,06E+08	1,09E+08	9,06E+07	1,35E+07	1,33E+08	0,00E+00
Polymeric immunoglobulin receptor (P81265 · PIGR_BOVIN)	110	117	CGLGISSR	0,00E+00	6,69E+07	2,25E+07	0,00E+00	0,00E+00	0,00E+00	0,00E+00	0,00E+00	0,00E+00
Polymeric immunoglobulin receptor (P81265 · PIGR_BOVIN)	147	156	TVTINCPFTR	0,00E+00	4,44E+06	4,12E+07	9,43E+06	3,89E+06	0,00E+00	0,00E+00	1,05E+07	0,00E+00
Polymeric immunoglobulin receptor (P81265 · PIGR_BOVIN)	251	268	SSVTFDCSLGPEVANVPK	7,82E+07	9,10E+07	7,71E+07	5,67E+07	5,46E+07	5,37E+07	0,00E+00	6,97E+07	0,00E+00
Polymeric immunoglobulin receptor (P81265 · PIGR_BOVIN)	274	288	KNGGACNVVINTLGK	4,32E+08	3,92E+08	4,48E+08	2,72E+08	2,32E+08	1,58E+08	1,78E+07	2,97E+08	0,00E+00
Polymeric immunoglobulin receptor (P81265 · PIGR_BOVIN)	275	288	NGGACNVVINTLGK	2,90E+08	2,79E+08	3,27E+08	2,50E+08	2,23E+08	1,51E+08	1,32E+07	2,85E+08	0,00E+00
Polymeric immunoglobulin receptor (P81265 · PIGR_BOVIN)	290	296	AQDFQGR	8,38E+06	4,81E+06	7,23E+06	9,30E+06	1,63E+07	6,16E+06	0,00E+00	2,06E+07	0,00E+00
Polymeric immunoglobulin receptor (P81265 · PIGR_BOVIN)	303	315	DNGVFSVHITSLR	5,19E+07	6,73E+07	8,00E+07	3,71E+07	3,32E+07	3,15E+07	0,00E+00	6,03E+07	0,00E+00
Polymeric immunoglobulin receptor (P81265 · PIGR_BOVIN)	363	375	GGSVTVSCPYNPK	8,10E+08	8,05E+08	8,26E+08	6,23E+08	5,45E+08	4,72E+08	5,02E+07	5,41E+08	0,00E+00
Polymeric immunoglobulin receptor (P81265 · PIGR_BOVIN)	382	393	YWCHWEEAQNGR	0,00E+00	0,00E+00	0,00E+00	0,00E+00	9,40E+05	3,28E+06	2,20E+07	0,00E+00	0,00E+00
Polymeric immunoglobulin receptor (P81265 · PIGR_BOVIN)	448	455	WISTVELK	0,00E+00	0,00E+00	0,00E+00	0,00E+00	0,00E+00	8,37E+06	7,71E+07	0,00E+00	0,00E+00
Polymeric immunoglobulin receptor (P81265 · PIGR_BOVIN)	456	464	VVQGEPSLK	2,93E+08	3,44E+08	7,34E+07	2,54E+08	8,54E+07	2,10E+08	6,59E+07	2,45E+08	0,00E+00
Polymeric immunoglobulin receptor (P81265 · PIGR_BOVIN)	479	486	LSCHFPCK	5,52E+07	4,79E+07	4,58E+07	5,11E+07	5,12E+07	3,34E+07	5,22E+06	4,25E+07	0,00E+00
Polymeric immunoglobulin receptor (P81265 · PIGR_BOVIN)	501	535	GCSALPTQNDGPSQAFVSCDQNSQVVSLNLDTVTK	0,00E+00	0,00E+00	0,00E+00	0,00E+00	0,00E+00	0,00E+00	0,00E+00	7,69E+05	0,00E+00
Polymeric immunoglobulin receptor (P81265 · PIGR_BOVIN)	536	546	EDEGWYWCGVK	8,35E+07	9,26E+07	9,04E+07	7,04E+07	6,25E+07	5,47E+07	0,00E+00	6,70E+07	0,00E+00

Polymeric immunoglobulin receptor (P81265 · PIGR_BOVIN)	551	564	YGETAAVYVAVESR	1,06E+08	1,19E+08	7,71E+07	6,30E+07	5,70E+07	6,90E+07	2,05E+06	9,12E+07	0,00E+00
Polymeric immunoglobulin receptor (P81265 · PIGR_BOVIN)	576	586	AAPAGAAIQSR	1,22E+08	1,40E+08	9,51E+07	1,02E+08	1,13E+08	8,92E+07	3,17E+07	1,01E+08	0,00E+00
Polymeric immunoglobulin receptor (P81265 · PIGR_BOVIN)	594	603	ALLDPSFFAK	0,00E+00	0,00E+00	0,00E+00	0,00E+00	0,00E+00	6,13E+06	0,00E+00	0,00E+00	0,00E+00
Polymeric immunoglobulin receptor (P81265 · PIGR_BOVIN)	608	630	DAAGGPGAPADPGRPTGYSGSSK	0,00E+00	0,00E+00	2,53E+07	1,72E+07	0,00E+00	4,68E+06	8,11E+06	2,44E+06	9,01E+07
Serotransferrin (Q29443 · TRFE_BOVIN)	24	37	TVRWCTISTHEANK	0,00E+00	0,00E+00	0,00E+00	0,00E+00	0,00E+00	0,00E+00	0,00E+00	0,00E+00	0,00E+00
Serotransferrin (Q29443 · TRFE_BOVIN)	27	37	WCTISTHEANK	0,00E+00	0,00E+00	0,00E+00	0,00E+00	0,00E+00	0,00E+00	1,33E+07	0,00E+00	0,00E+00
Serotransferrin (Q29443 · TRFE_BOVIN)	107	120	DNPQTHYYAVAVVK	0,00E+00	1,23E+07	1,63E+07	8,90E+07	7,90E+07	7,82E+07	0,00E+00	1,01E+08	0,00E+00
Serotransferrin (Q29443 · TRFE_BOVIN)	156	166	ELPDPQESIQR	1,32E+07	1,52E+07	1,40E+07	3,43E+07	3,24E+07	2,93E+07	1,31E+06	4,34E+07	0,00E+00
Serotransferrin (Q29443 · TRFE_BOVIN)	230	243	HSTVFDNLPNPEDR	0,00E+00	0,00E+00	0,00E+00	2,43E+07	1,14E+06	1,30E+07	0,00E+00	1,26E+07	0,00E+00
Serotransferrin (Q29443 · TRFE_BOVIN)	245	255	NYELLCGDNTR	3,60E+07	3,97E+07	3,77E+07	5,83E+07	2,40E+07	6,19E+07	1,05E+07	7,56E+07	0,00E+00
Serotransferrin (Q29443 · TRFE_BOVIN)	283	299	EDVIWELLNHAQEHFGK	0,00E+00	0,00E+00	0,00E+00	4,16E+06	2,07E+06	3,38E+06	0,00E+00	1,01E+07	0,00E+00
Serotransferrin (Q29443 · TRFE_BOVIN)	300	314	DKPDNFQLFQSPHGK	0,00E+00	0,00E+00	0,00E+00	1,39E+07	0,00E+00	7,27E+06	0,00E+00	2,31E+07	5,64E+07
Serotransferrin (Q29443 · TRFE_BOVIN)	320	327	DSADGFLK	7,78E+06	1,29E+07	1,02E+07	3,23E+07	4,13E+07	3,55E+07	0,00E+00	4,79E+07	0,00E+00
Serotransferrin (Q29443 · TRFE_BOVIN)	366	374	WCAIGHQER	4,04E+07	3,76E+07	3,24E+07	4,52E+07	4,94E+07	3,18E+07	9,71E+06	3,58E+07	0,00E+00
Serotransferrin (Q29443 · TRFE_BOVIN)	424	435	CGLVPVLAENYK	0,00E+00	3,22E+06	0,00E+00	0,00E+00	0,00E+00	0,00E+00	0,00E+00	0,00E+00	0,00E+00
Serotransferrin (Q29443 · TRFE_BOVIN)	448	456	GYLAVAVVK	2,25E+08	2,19E+08	2,58E+08	1,97E+08	1,81E+08	1,69E+08	5,32E+07	2,26E+08	5,45E+07
Serotransferrin (Q29443 · TRFE_BOVIN)	499	513	FDEFFSAGCAPGSPR	1,05E+08	1,08E+08	1,04E+08	9,56E+07	9,34E+07	7,73E+07	2,54E+07	9,44E+07	0,00E+00
Serotransferrin (Q29443 · TRFE_BOVIN)	531	539	ECVPNSNER	3,65E+06	5,84E+05	0,00E+00	0,00E+00	0,00E+00	0,00E+00	0,00E+00	0,00E+00	0,00E+00
Serotransferrin (Q29443 · TRFE_BOVIN)	561	578	DQTVIQNTDGNNNEAWAK	0,00E+00	0,00E+00	0,00E+00	0,00E+00	0,00E+00	0,00E+00	1,13E+07	0,00E+00	0,00E+00
Serotransferrin (Q29443 · TRFE_BOVIN)	583	590	ENFEVLCK	0,00E+00	0,00E+00	0,00E+00	0,00E+00	0,00E+00	0,00E+00	3,91E+07	0,00E+00	0,00E+00
Serotransferrin (Q29443 · TRFE_BOVIN)	595	607	KPVTDAENCHLAR	0,00E+00	0,00E+00	0,00E+00	0,00E+00	5,82E+06	1,22E+07	4,57E+07	0,00E+00	0,00E+00
Serotransferrin (Q29443 · TRFE_BOVIN)	671	682	TYDSYLGDDYVR	9,12E+07	6,29E+07	6,01E+07	6,07E+07	5,90E+07	5,93E+07	3,10E+07	7,44E+07	2,96E+07

Serotransferrin (Q29443 · TRFE_BOVIN)	695	704	LLEACTFHKP	0,00E+00	0,00E+00	1,33E+07	1,44E+07	9,42E+06	5,76E+06	0,00E+00	1,06E+07	0,00E+00
Serotransferrin (Q29443 · TRFE_BOVIN; Q0PGA7_BUBBU)	133	140	SCHTGLGR	4,03E+06	0,00E+00	0,00E+00	0,00E+00	3,15E+06	0,00E+00	5,83E+06	4,40E+06	0,00E+00
Serotransferrin (Q29443 · TRFE_BOVIN; Q0PGA7_BUBBU)	475	482	SCHTAVDR	0,00E+00	0,00E+00	0,00E+00	0,00E+00	0,00E+00	0,00E+00	0,00E+00	1,37E+06	0,00E+00
Serotransferrin (Q29443 · TRFE_BOVIN; Q0PGA7_BUBBU)	542	550	YYGYTGAFR	4,78E+08	3,33E+08	5,98E+08	4,58E+08	4,37E+08	4,91E+08	3,70E+08	5,40E+08	2,75E+08
Serum amyloid A protein (V5LEH6_BUBBU)	66	74	GPGGAWAAK	0,00E+00	0,00E+00	0,00E+00	0,00E+00	0,00E+00	0,00E+00	9,56E+06	0,00E+00	0,00E+00
Serum amyloid A protein (V5LEH6_BUBBU)	81	92	ETIQGITDPLLK	1,04E+07	1,32E+07	1,13E+07	1,33E+07	1,39E+07	1,26E+07	0,00E+00	1,27E+07	2,80E+07
Serum amyloid A protein (V5LEH6_BUBBU)	105	114	ADQFANEWGR	0,00E+00	0,00E+00	0,00E+00	0,00E+00	6,73E+06	1,33E+07	8,09E+06	0,00E+00	0,00E+00
Vitamin D-binding protein (Q3MHN5 · VTDB_BOVIN)	95	119	SCESNSPFPVHPGTPECCTHEGLEK	3,33E+07	3,19E+07	2,93E+07	2,14E+07	2,07E+07	2,40E+07	3,63E+07	2,38E+07	0,00E+00
Vitamin D-binding protein (Q3MHN5 · VTDB_BOVIN)	128	149	HQPQEFPTYVEPTNDEICEAFR	0,00E+00	0,00E+00	0,00E+00	0,00E+00	0,00E+00	0,00E+00	8,13E+06	3,81E+06	0,00E+00
Vitamin D-binding protein (Q3MHN5 · VTDB_BOVIN)	219	228	ICSQYAAYGK	0,00E+00	0,00E+00	0,00E+00	0,00E+00	0,00E+00	0,00E+00	2,34E+06	0,00E+00	0,00E+00
Vitamin D-binding protein (Q3MHN5 · VTDB_BOVIN)	276	283	ELPEYAVK	1,89E+07	2,05E+07	1,86E+07	1,09E+07	1,90E+07	1,59E+07	2,80E+07	1,60E+07	0,00E+00
Vitamin D-binding protein (Q3MHN5 · VTDB_BOVIN)	284	291	LCDNLSTK	2,00E+07	1,16E+07	1,23E+07	0,00E+00	0,00E+00	0,00E+00	0,00E+00	2,04E+07	0,00E+00
Vitamin D-binding protein (Q3MHN5 · VTDB_BOVIN)	341	351	VLDQYIFELSR	4,62E+06	4,40E+06	4,14E+06	1,12E+06	2,87E+06	3,27E+06	3,16E+06	1,13E+06	0,00E+00
Vitamin D-binding protein (Q3MHN5 · VTDB_BOVIN)	352	362	KTQIPEVFLTK	0,00E+00	0,00E+00	1,35E+06	0,00E+00	0,00E+00	0,00E+00	0,00E+00	0,00E+00	0,00E+00
Vitamin D-binding protein (Q3MHN5 · VTDB_BOVIN)	363	369	ILESTLK	3,32E+07	3,77E+07	3,61E+07	0,00E+00	0,00E+00	0,00E+00	0,00E+00	2,47E+07	0,00E+00
Vitamin D-binding protein (Q3MHN5 · VTDB_BOVIN)	370	387	SLDECCHSESSTACLNAK	3,92E+07	4,19E+07	3,63E+07	3,57E+07	3,48E+07	3,82E+07	4,07E+07	2,66E+07	0,00E+00
Vitamin D-binding protein (Q3MHN5 · VTDB_BOVIN)	394	401	ELSSFIQK	2,51E+07	2,95E+07	1,16E+07	0,00E+00	0,00E+00	6,85E+06	2,74E+07	0,00E+00	0,00E+00
Vitamin D-binding protein (Q3MHN5 · VTDB_BOVIN)	402	418	GQELCADYSENTFTEYK	3,60E+06	1,56E+06	4,11E+06	8,77E+05	1,67E+06	1,14E+06	2,83E+06	1,27E+06	0,00E+00
Vitamin D-binding protein (Q3MHN5 · VTDB_BOVIN)	429	443	FPDATETDLQELVAK	0,00E+00	0,00E+00	0,00E+00	0,00E+00	0,00E+00	0,00E+00	0,00E+00	0,00E+00	0,00E+00
Vitamin K-dependent protein S (P07224 · PROS_BOVIN)	474	481	HCLVNVEK	1,15E+06	1,66E+06	5,05E+05	8,09E+05	7,94E+05	0,00E+00	0,00E+00	6,83E+05	0,00E+00

Xanthine dehydrogenase/oxidase (P80457 · XDH_BOVIN)	451	462	ELALCYGGMADR	0,00E+00	0,00E+00	0,00E+00	1,20E+06	1,43E+06	0,00E+00	0,00E+00	0,00E+00	0,00E+00
Xanthine dehydrogenase/oxidase (P80457 · XDH_BOVIN)	974	980	SSQYYAR	0,00E+00	0,00E+00	0,00E+00	0,00E+00	0,00E+00	0,00E+00	1,83E+06	0,00E+00	0,00E+00
Xanthine dehydrogenase/oxidase (G1AQP3_BUBBU)	856	871	VVALEVDHYSNAGNSR	5,52E+06	2,84E+06	3,23E+06	2,80E+07	9,94E+06	1,84E+07	0,00E+00	1,37E+07	0,00E+00
Xanthine dehydrogenase/oxidase (P80457 · XDH_BOVIN; G1AQP3_BUBBU)	2	13	TADELVFFVNGK	0,00E+00	0,00E+00	0,00E+00	0,00E+00	0,00E+00	0,00E+00	0,00E+00	9,82E+05	0,00E+00
Xanthine dehydrogenase/oxidase (P80457 · XDH_BOVIN; G1AQP3_BUBBU)	19	31	NADPETTLLAYLR	0,00E+00	0,00E+00	0,00E+00	0,00E+00	0,00E+00	0,00E+00	0,00E+00	2,79E+05	0,00E+00
Xanthine dehydrogenase/oxidase (P80457 · XDH_BOVIN; G1AQP3_BUBBU)	234	243	VTWIQASTLK	0,00E+00	0,00E+00	0,00E+00	1,56E+07	1,54E+07	1,77E+07	1,62E+07	2,12E+07	0,00E+00
Xanthine dehydrogenase/oxidase (P80457 · XDH_BOVIN; G1AQP3_BUBBU)	329	335	GVLEQLR	3,88E+06	3,08E+06	4,59E+06	2,78E+07	2,27E+07	2,32E+07	1,84E+07	2,91E+07	0,00E+00
Xanthine dehydrogenase/oxidase (P80457 · XDH_BOVIN; G1AQP3_BUBBU)	414	422	EDEFFSAFK	0,00E+00	0,00E+00	0,00E+00	0,00E+00	0,00E+00	0,00E+00	0,00E+00	1,40E+06	0,00E+00
Xanthine dehydrogenase/oxidase (P80457 · XDH_BOVIN; G1AQP3_BUBBU)	519	525	FYLTVLK	0,00E+00	0,00E+00	0,00E+00	1,44E+06	0,00E+00	1,64E+06	1,74E+06	1,67E+06	0,00E+00
Xanthine dehydrogenase/oxidase (P80457 · XDH_BOVIN; G1AQP3_BUBBU)	538	551	LDPTYTSATLLFQK	0,00E+00	0,00E+00	0,00E+00	0,00E+00	0,00E+00	0,00E+00	0,00E+00	6,40E+05	0,00E+00
Xanthine dehydrogenase/oxidase (P80457 · XDH_BOVIN; G1AQP3_BUBBU)	599	606	YENELFLR	0,00E+00	0,00E+00	0,00E+00	3,89E+06	3,57E+06	4,58E+06	1,06E+07	0,00E+00	0,00E+00
Xanthine dehydrogenase/oxidase (P80457	779	786	MLGVPVNR	0,00E+00	0,00E+00	0,00E+00	0,00E+00	0,00E+00	0,00E+00	0,00E+00	2,57E+06	0,00E+00

· XDH_BOVIN; G1AQP3_BUBBU)												
Xanthine dehydrogenase/oxidase (P80457 · XDH_BOVIN; G1AQP3_BUBBU)	805	818	STLVSVAVALAAYK	0,00E+00	0,00E+00	0,00E+00	0,00E+00	0,00E+00	9,76E+05	0,00E+00	5,33E+05	0,00E+00
Xanthine dehydrogenase/oxidase (P80457 · XDH_BOVIN; G1AQP3_BUBBU)	830	839	NEDMLITGGR	0,00E+00	0,00E+00	0,00E+00	1,78E+06	0,00E+00	0,00E+00	0,00E+00	2,90E+06	0,00E+00
Xanthine dehydrogenase/oxidase (P80457 · XDH_BOVIN; G1AQP3_BUBBU)	881	890	ALFHMDNCYK	0,00E+00	0,00E+00	0,00E+00	9,40E+06	8,45E+06	7,57E+06	0,00E+00	1,13E+07	0,00E+00
Xanthine dehydrogenase/oxidase (P80457 · XDH_BOVIN; G1AQP3_BUBBU)	903	912	TNLSSNTAFR	7,29E+06	7,95E+06	9,83E+06	3,20E+07	3,09E+07	3,05E+07	1,90E+07	2,83E+07	0,00E+00
Xanthine dehydrogenase/oxidase (P80457 · XDH_BOVIN; G1AQP3_BUBBU)	949	958	EGDLTHFNQR	0,00E+00	0,00E+00	0,00E+00	0,00E+00	0,00E+00	0,00E+00	0,00E+00	3,85E+06	0,00E+00
Xanthine dehydrogenase/oxidase (P80457 · XDH_BOVIN; G1AQP3_BUBBU)	959	966	LEGFSVPR	0,00E+00	0,00E+00	0,00E+00	0,00E+00	0,00E+00	0,00E+00	0,00E+00	0,00E+00	0,00E+00
Xanthine dehydrogenase/oxidase (P80457 · XDH_BOVIN; G1AQP3_BUBBU)	1125	1134	VSLSTTGFYR	0,00E+00	0,00E+00	0,00E+00	1,62E+07	2,11E+07	2,18E+07	2,11E+07	2,36E+07	0,00E+00
Xanthine dehydrogenase/oxidase (P80457 · XDH_BOVIN; G1AQP3_BUBBU)	1229	1240	IPAFGSIPTEFR	4,22E+05	0,00E+00	8,48E+05	0,00E+00	0,00E+00	0,00E+00	3,28E+06	2,03E+06	0,00E+00
Xanthine dehydrogenase/oxidase (P80457 · XDH_BOVIN; G1AQP3_BUBBU)	1296	1304	LDSPATPEK	0,00E+00	0,00E+00	0,00E+00	1,38E+06	0,00E+00	0,00E+00	0,00E+00	1,73E+06	0,00E+00

Supplementary Material Figure S1.

MALDI-TOF-MS analysis of pasteurized buffalo milk spiked with different volumes of the bovine counterpart. The pH 4.6 soluble protein fraction of each sample was extracted and subjected to trypsinolysis as reported in the experimental section. Resulting peptides were subjected to mass spectrometric analysis. Calibration curve was obtained by plotting the signal intensity of the bovine proteotypic peptide β-Lg (f149-162) at the different percentage adulteration values. The observed values are derived from the average of three observations for each point and are reported ± S.D.

Species	Peptide	MH+	% Bovine milk		
			1%	3%	5%
Bovine	β-Lg (f149-162)	1657,8	0,04±0,01	0,06±0,01	0,08±0,02

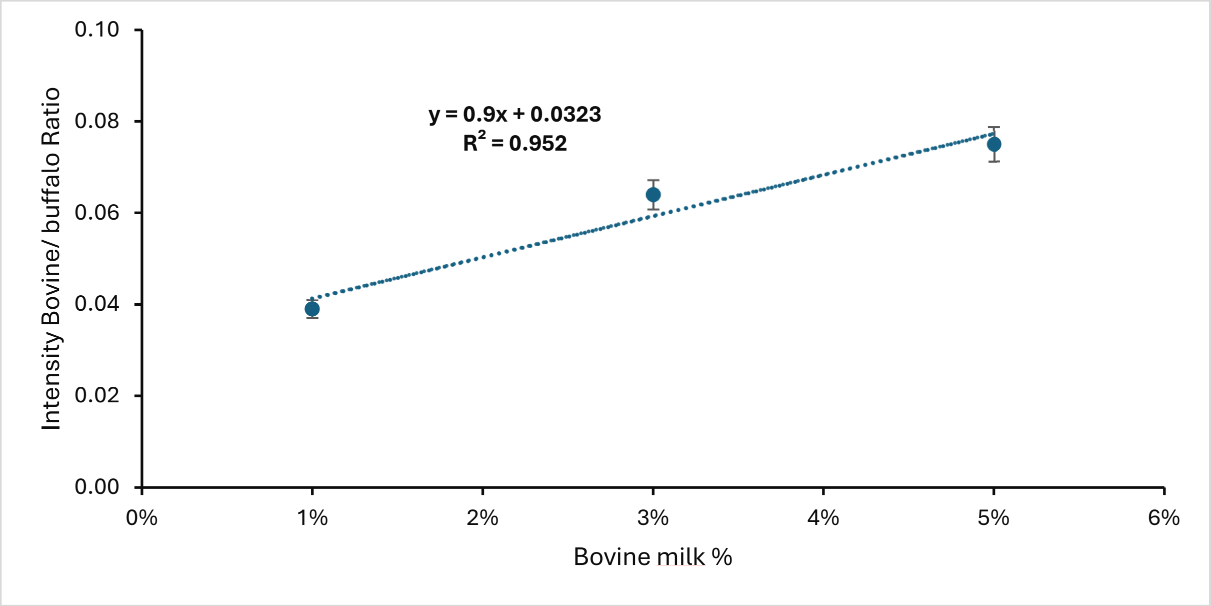

Supplementary Material Figure S2.

MALDI-TOF-MS analysis of pasteurized buffalo milk spiked with different volumes of the bovine counterpart. The pH 4.6 soluble protein fraction of each sample was extracted and subjected to trypsinolysis as reported in the experimental section. Resulting peptides were subjected to mass spectrometric analysis. Calibration curve was obtained by plotting the signal intensity of the bovine proteotypic peptide β-Lg (f149-162) at the different percentage adulteration values. The observed values are derived from the average of three observations for each point and are reported ± S.D.

Species	Peptide	MH^+	% Bovine milk		
			10%	20%	30%
Bovine	β-Lg (f149-162)	1657,8	0,16±0,01	0,33±0,03	0,43±0,02

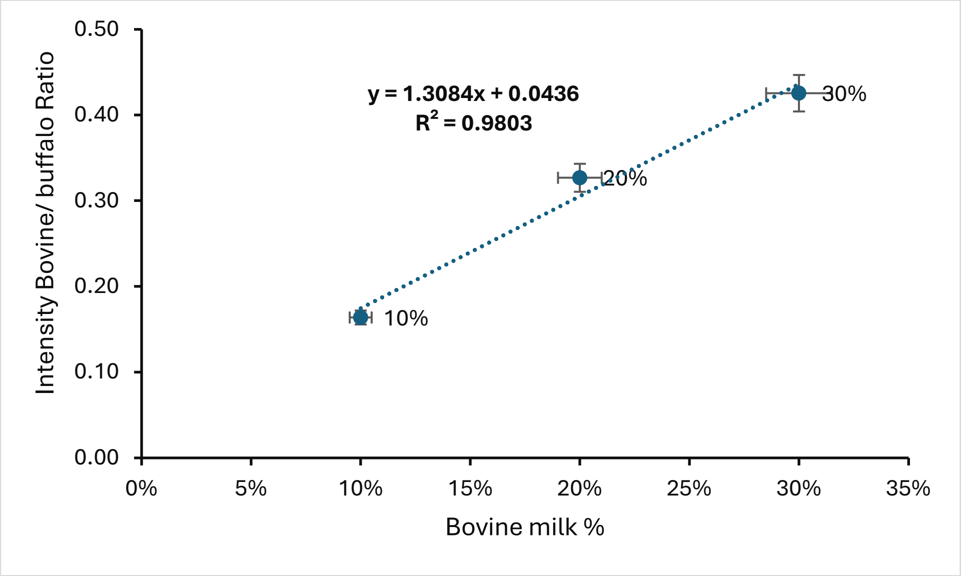

Supplementary Material Figure S3.

MALDI-TOF-MS analysis of pasteurized buffalo milk spiked with different volumes of the bovine counterpart. The pH 4.6 soluble protein fraction of each sample was extracted and subjected to trypsinolysis as reported in the experimental section. Resulting peptides were subjected to mass spectrometric analysis. Calibration curve was obtained by plotting the signal intensity of the bovine proteotypic carboxymethylated peptide α-La (f17-58) at the different percentage adulteration values. The observed values are derived from the average of three observations for each point and are reported ± S.D.

Species	Peptide	MH^+	% Bovine milk		
			10%	20%	30%
Bovine	α-La (f17-58)	4653,14	0,02±0,02	0,16±0,00	0,27±0,15

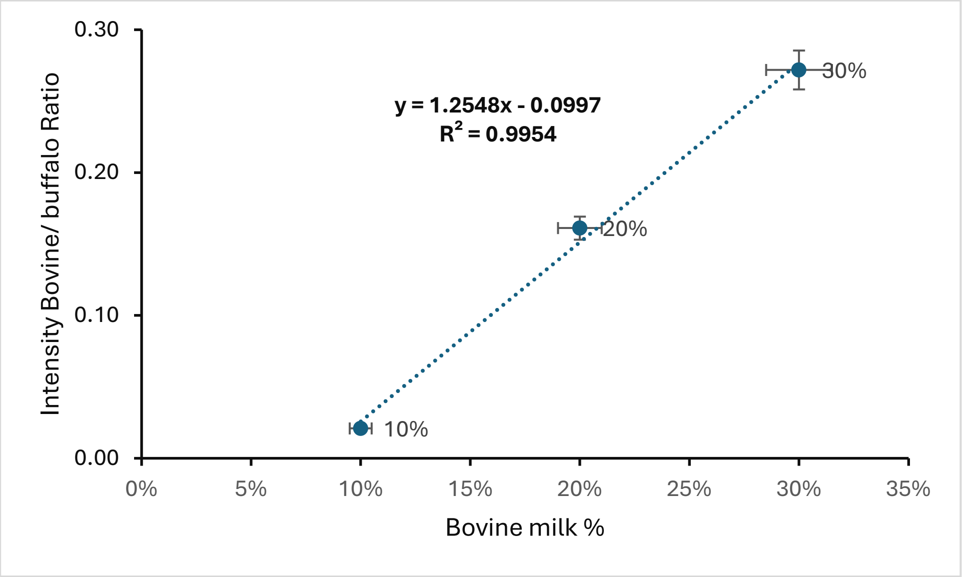

Supplementary Material Figure S4.

MALDI-TOF-MS analysis of pasteurized buffalo milk spiked with different volumes of the bovine counterpart. The pH 4.6 soluble protein fraction of each sample was extracted and subjected to trypsinolysis as reported in the experimental section. Resulting peptides were subjected to mass spectrometric analysis. Calibration curve was obtained by plotting the signal intensity of the bovine proteotypic carboxymethylated Cys_{28} peptide α-La (f17-58) at the different percentage adulteration values. The observed values are derived from the average of three observations for each point and are reported ± S.D.

Species	Peptide	MH+	% Bovine milk		
			1%	3%	5%
Bovine	α-La (f17-58)	4711,1	0,01±0,00	0,01±0,00	0,02±0,01

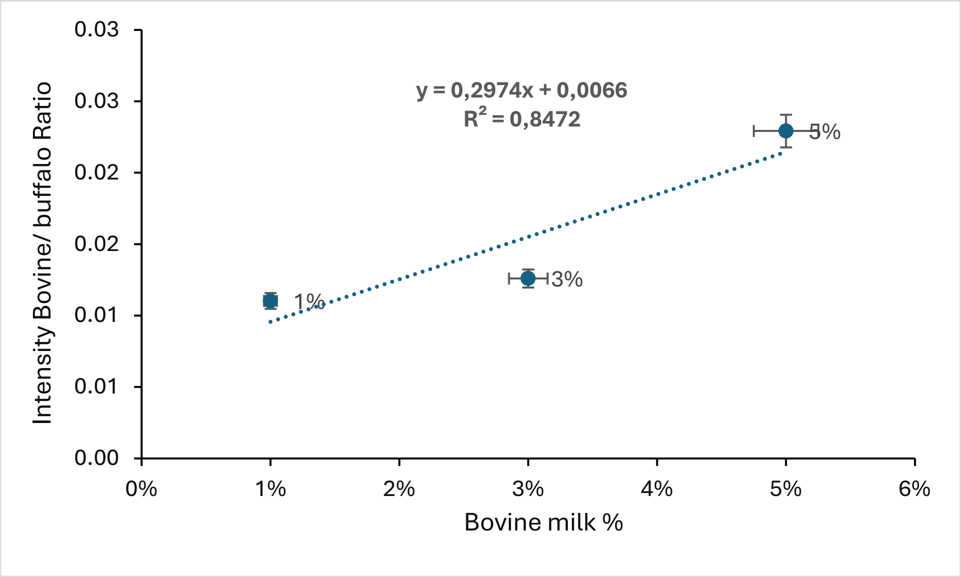

Supplementary Material Figure S5.

Nano-HPLC-ESI-MS/MS analysis of pasteurized buffalo milk spiked with different volumes of the bovine counterpart. The pH 4.6 soluble protein fraction of each sample was extracted and subjected to trypsinolysis as reported in the experimental section. Resulting peptides were subjected to nano-HPLC-ESI-MS/MS. Calibration curve was obtained by plotting the signal intensity of the bovine proteotypic carboxymethylated β-Lg (f149-162) at the different percentage adulteration values. The observed values are derived from the average of three observations for each point and are reported ± S.D.

Species	Peptide	% Bovine milk								
		MH+	0%	1%	3%	5%	10%	20%	30%	100%
Bovine	β-Lg (f149-162)	1714,8	0,00	1,16	2,43	3,42	7,98	18,09	25,52	98,96

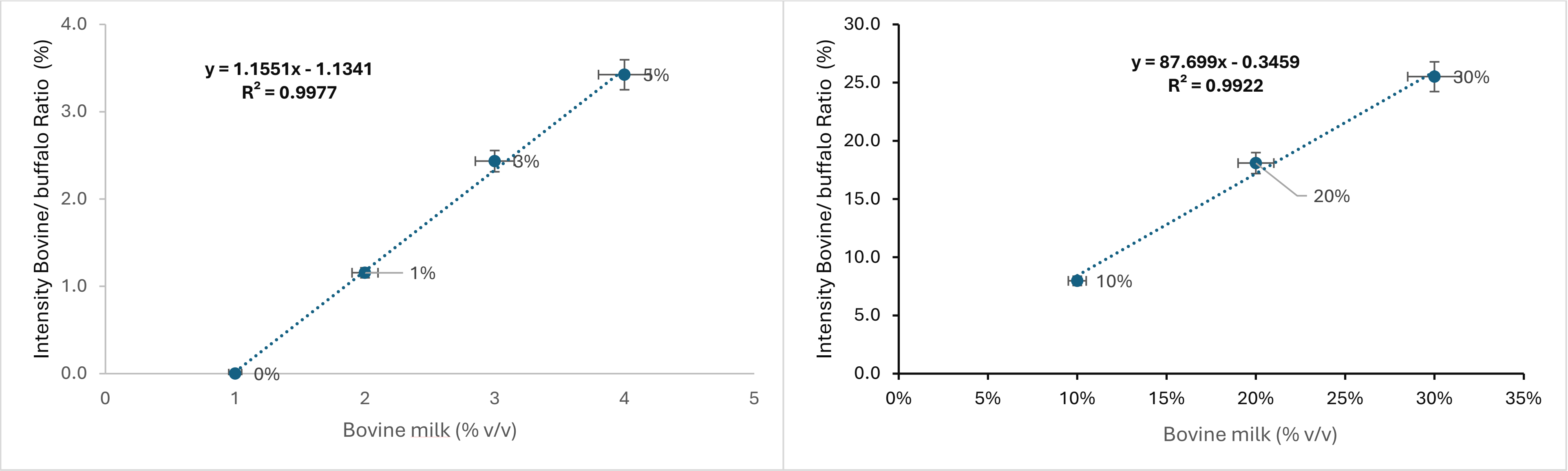

Supplement: Supplementary file 1 [file foods-14-00822-s001.zip › foods-3458908-supplementary.pdf]
